# Cervical cerclage for prevention of preterm birth and adverse perinatal outcome in twin pregnancies with short cervical length or cervical dilatation: A systematic review and meta-analysis

**DOI:** 10.1371/journal.pmed.1004266

**Published:** 2023-08-03

**Authors:** Francesco D’Antonio, Nashwa Eltaweel, Smriti Prasad, Maria Elena Flacco, Lamberto Manzoli, Asma Khalil

**Affiliations:** 1 Center for Fetal Care and High-Risk Pregnancy, University of Chieti, Chieti, Italy; 2 Division of Biomedical Science, Warwick Medical School University of Warwick, University Hospital of Coventry and Warwickshire, Coventry, United Kingdom; 3 Fetal Medicine Unit, St George’s Hospital, London, United Kingdom; 4 Department of Environmental and Preventive Sciences, University of Ferrara, Ferrara, Italy; 5 Department of Medical and Surgical Sciences, University of Bologna, Bologna, Italy; 6 Vascular Biology Research Centre, Molecular and Clinical Sciences Research Institute, St George’s University of London, London, United Kingdom; 7 Twins Trust Centre for Research and Clinical Excellence, St George’s Hospital, London, United Kingdom; 8 Fetal Medicine Unit, Liverpool Women’s Hospital, University of Liverpool, Liverpool, United Kingdom

## Abstract

**Background:**

The optimal approach to prevent preterm birth (PTB) in twins has not been fully established yet. Recent evidence suggests that placement of cervical cerclage in twin pregnancies with short cervical length at ultrasound or cervical dilatation at physical examination might be associated with a reduced risk of PTB. However, such evidence is based mainly on small studies thus questioning the robustness of these findings. The aim of this systematic review was to determine the role of cervical cerclage in preventing PTB and adverse maternal or perinatal outcomes in twin pregnancies.

**Methods and findings:**

Key databases searched and date of last search: MEDLINE, Embase, and CINAHL were searched electronically on 20 April 2023.

Eligibility criteria: Inclusion criteria were observational studies assessing the risk of PTB among twin pregnancies undergoing cerclage versus no cerclage and randomized trials in which twin pregnancies were allocated to cerclage for the prevention of PTB or to a control group (e.g., placebo or treatment as usual). The primary outcome was PTB <34 weeks of gestation. The secondary outcomes were PTB <37, 32, 28, 24 weeks of gestation, gestational age at birth, the interval between diagnosis and birth, preterm prelabor rupture of the membranes (pPROM), chorioamnionitis, perinatal loss, and perinatal morbidity. Subgroup analyses according to the indication for cerclage (short cervical length or cervical dilatation) were also performed.

Risk of bias assessment: The risk of bias of the included randomized controlled trials (RCTs) was assessed using the Revised Cochrane risk-of-bias tool for randomized trials, while that of the observational studies using the Newcastle–Ottawa scale (NOS).

Statistical analysis: Summary risk ratios (RRs) of the likelihood of detecting each categorical outcome in exposed versus unexposed women, and (b) summary mean differences (MDs) between exposed and unexposed women (for each continuous outcome), with their 95% confidence intervals (CIs) were computed using head-to-head meta-analyses.

Synthesis of the results: Eighteen studies (1,465 twin pregnancies) were included. Placement of cervical cerclage in women with a twin pregnancy with a short cervix at ultrasound or cervical dilatation at physical examination was associated with a reduced risk of PTB <34 weeks of gestation (RR: 0.73, 95% CI [0.59, 0.91], *p* = 0.005 corresponding to a 16% difference in the absolute risk, AR), <32 (RR: 0.69, 95% CI [0.57, 0.84], *p* < 0.001; AR: 16.92%), <28 (RR: 0.54, 95% [CI 0.43, 0.67], 0.001; AR: 18.29%), and <24 (RR: 0.48, 95% CI [0.23, 0.97], *p* = 0.04; AR: 15.57%) weeks of gestation and a prolonged gestational age at birth (MD: 2.32 weeks, 95% [CI 0.99, 3.66], *p* < 0.001). Cerclage in twin pregnancy with short cervical length or cervical dilatation was also associated with a reduced risk of perinatal loss (RR: 0.38, 95% CI [0.25, 0.60], *p* < 0.001; AR: 19.62%) and composite adverse outcome (RR: 0.69, 95% CI [0.53, 0.90], *p* = 0.007; AR: 11.75%). Cervical cerclage was associated with a reduced risk of PTB <34 weeks both in women with cervical length <15 mm (RR: 0.74, 95% CI [0.58, 0.95], *p* = 0.02; AR: 29.17%) and in those with cervical dilatation (RR: 0.68, 95% CI [0.57, 0.80], *p* < 0.001; AR: 35.02%). The association between cerclage and prevention of PTB and adverse perinatal outcomes was exclusively due to the inclusion of observational studies. The quality of retrieved evidence at GRADE assessment was low.

**Conclusions:**

Emergency cerclage for cervical dilation or short cervical length <15 mm may be potentially associated with a reduction in PTB and improved perinatal outcomes. However, these findings are mainly based upon observational studies and require confirmation in large and adequately powered RCTs.

## Abstract 2

In a systematic review and meta-analysis, Francesco D’Antonio and colleagues investigate the impact of cervical cerclage on preterm birth in women with twin pregnancy.

## Author summary

**Why was this study done?:**

- Twin pregnancies are at high risk of preterm birth (PTB).
- Recent evidence suggests that placement of cervical cerclage in twin pregnancies with short cervical length at ultrasound or cervical dilatation at physical examination might be associated with a reduced risk of PTB.
- However, such evidence is based mainly on small studies thus questioning the robustness of these findings.

**What did the researchers do and find?:**

- We performed a systematic review and meta-analysis to elucidate whether cervical cerclage in women with twin pregnancy with short cervical length or cervical dilatation may prevent PTB.
- We included 18 studies. The primary outcome was PTB <34 weeks of gestation.
- We found that cervical cerclage in women with short cervical length or cervical dilatation was associated with a reduced risk of PTB <34 weeks, gestational age at birth, and adverse neonatal outcome.
- The strength of association between cerclage and reduced risk of PTB was maintained when considering women with short cervix on ultrasound and those with cervical dilatation at physical examination separately.

**What do these findings mean?:**

- Cervical cerclage in twin pregnancies with short cervical length or cervical dilatation may be potentially associated with a reduced risk of PTB and improved neonatal outcomes.
- However, these findings are mainly based on observational studies and, to improve robustness of evidence, confirmation of these outcomes in large and appropriately designed randomized controlled trials (RCTs) is required.

## Introduction

Twin pregnancies are at increased risk of perinatal morbidity and mortality compared to singletons, primarily due to preterm birth (PTB), fetal anomalies, and complications unique to monochorionic (MC) placenta, such as twin-to-twin transfusion syndrome (TTTS) and selective fetal growth restriction (sFGR) [1–8]. The incidence of PTB in twin pregnancy has been reported to be approximately 20% in recent series and this risk differs according to the chorionicity and amnionicity. Around 60% of twin pregnancies deliver prior to 37 weeks and 12% before 34 weeks of gestation, with rates 5 and 8 times higher than the equivalent rates for a singleton pregnancy, respectively [9].

In singleton pregnancies with recognized risk factors for PTB, vaginal progesterone is the primary intervention with consistently demonstrated effectiveness in preventing PTB, followed by cervical cerclage [10]. However, observational studies and systematic reviews have reported a beneficial role of cervical cerclage in pregnancies with an extremely short cervix, defined as a cervical length of less than 10 mm on ultrasound scan [11].

Conversely, there is less evidence on the optimal strategy for preventing PTB in twin pregnancies. Several randomized trials and systematic reviews reported little or no benefit of vaginal progesterone, cerclage, or pessary in twin pregnancies [12–14]. However, these studies were limited by small sample size and large heterogeneity in their inclusion criteria, study populations, and outcomes observed. These limitations did not allow the authors to reach evidence-based conclusions on the role of these interventions in reducing the risk of PTB in twin pregnancies. More importantly, in the last few years, an increasing number of studies reporting a potential beneficial role of cerclage in reducing the risk of PTB and adverse outcomes in twin pregnancies have been published [15–20]. These studies have challenged the prevailing view around the lack of effectiveness of cerclage in twin pregnancies.

We performed a systematic review and meta-analysis of the published literature to determine the role of cervical cerclage in preventing PTB and adverse maternal and perinatal outcomes in twin pregnancies.

## Methods

### Data sources

This review was performed according to an a priori designed protocol recommended for systematic reviews and meta-analysis [21–24]. MEDLINE, Embase, and CINAHL were searched electronically since inception on 6 July 2022 and updated on 20 April 2023 utilizing combinations of the relevant medical subject heading (MeSH) terms, keywords, and word variants for “twin pregnancies,” “multiple pregnancies,” “cerclage,” and “preterm birth.” The search and selection criteria with no language restriction. The search strategy is outlined in S1 Table. The reference lists of relevant articles and reviews were hand-searched for additional reports. The study was registered with the PROSPERO database (Registration number: CRD42022351058). This study is reported as per the Preferred Reporting Items for Systematic Reviews and Meta-Analyses (PRISMA) guidelines (S2 Table) [25].

### Eligibility criteria, main outcomes measures

Inclusion criteria were observational studies assessing the risk of PTB among twin pregnancies undergoing cerclage versus no cerclage and randomized trials in which twin pregnancies were allocated to cerclage for the prevention of PTB or to a control group (e.g., placebo or treatment as usual).

The primary outcome was PTB <34 weeks of gestation.

The secondary outcomes were:

- PTB <37 weeks
- PTB <32 weeks
- PTB <28 weeks
- PTB <24 weeks
- Gestational age at birth [weeks]
- Interval between diagnosis and birth [weeks]
- Preterm prelabor rupture of the membranes (pPROM), defined as the rupture of the membranes before labor and before 37 weeks of gestation
- Chorioamnionitis
- Perinatal loss, including miscarriage, intra-uterine, and neonatal death
- Apgar score <7 at 5 min
- Birthweight <2,500 grams
- Birthweight <1,500 grams
- Birthweight expressed as a continuous variable
- Respiratory distress syndrome (RDS)
- Intraventricular hemorrhage (IVH), grades III and IV
- Necrotizing enterocolitis (NEC)
- Retinopathy of prematurity (ROP)
- Neonatal sepsis
- Admission to the neonatal intensive care unit (NICU)
- Length of stay in NICU (days).

Both primary and secondary outcomes were explored first in women with either cervical dilatation at a physical examination or short cervix (<25 mm) at ultrasound and in those with short cervical length at ultrasound and cervical dilatation separately. Furthermore, we planned to perform subgroup analyses according to different cut-offs of cervical length at ultrasound (<25 mm, <15 mm, and <10 mm) and cervical dilatation at physical examination (<2 cm versus >2 cm), according to chorionicity and type of cerclage (McDonald versus Shirodkar). In the McDonald technique, a suture is placed around the cervix in purse-string fashion and securely tied anteriorly. Conversely, the Shirodkar technique requires a transverse incision in the vaginal mucosa of the anterior and posterior cervix to avoid injury of the bladder and rectum, respectively. The lateral angles of the anterior and posterior incisions are then expanded with blunt fingertip dissection of the lateral cervix and a woven thread is then passed through the submucosal tunnel from anterior to posterior on both sides of the cervix. After the suture is placed on both sides of the cervix, the knot is tied in the posterior defect.

### Data collection and analysis

Two reviewers (FDA, NA) independently extracted data. Inconsistencies were discussed among the reviewers and consensus reached. For those articles in which data on short cervical length was not reported separately for subgroups of women (<15 mm and 15 to 25 mm), but the methodology was such that the information might have been recorded initially, the authors were contacted, and the data requested.

The risk of bias of the included randomized controlled trial (RCTs) was assessed using the Revised Cochrane risk-of-bias tool for randomized trials (RoB 2) [26]. According to this tool, the risk of bias in each included study is judged according to 5 domains: bias arising from the randomization process, bias due to deviations from intended interventions, bias due to missing outcome data, bias in the measurement of the outcome, and bias in the selection of the reported result. Although the RoB2 tool does not provide an overall risk of bias assessment, the overall risk of bias was considered low if 4 or more domains were rated as low risk (not counting “other biases”), with at least one being sequence generation or allocation concealment, according to what was reported in previous systematic reviews of intervention.

The risk of bias in the observational studies was performed using the Newcastle–Ottawa scale (NOS) for cohort studies [27]. According to NOS, each study is judged on 3 broad perspectives: selection of the study groups, comparability of the groups, and ascertainment of the outcome of interest. Assessment of the selection of a study includes the evaluation of the representativeness of the exposed cohort, selection of the nonexposed cohort, ascertainment of exposure, and the demonstration that the outcome of interest was not present at the start of the study. Assessment of the comparability of the study includes the evaluation of the comparability of cohorts based on the design or analysis. Finally, ascertainment of the outcome of interest includes the evaluation of the type of assessment of the outcome of interest, and length and adequacy of follow-up. According to NOS, a study can be awarded a maximum of 1 star for each numbered item within the selection and outcome categories. A maximum of 2 stars can be given for comparability [27]. The conclusions of the meta-analysis on the primary outcome were assessed using the GRADE approach by the first author, who was familiar with GRADE (GRADEpro, Version 20, 2014, McMaster University, Hamilton, Ontario, Canada) [28]. A second author verified the ratings; any disagreements were reconciled after discussion. The pooled analysis of the primary outcome was assessed in relation to the quality of the evidence scored in the 5 domains specified within GRADE: limitations in study design and/or execution (risk of bias), inconsistency of results, indirectness of evidence, imprecision of results, and publication bias [28].

### Statistical analysis

We examined a total of 17 maternal and perinatal outcomes, either categorical or continuous, in a sample of women with twin pregnancies at risk of PTB undergoing cerclage (exposed women) versus no cerclage (unexposed women). All analyses were performed 3 times: (a) including women undergoing cerclage for either cervical dilatation or short cervical length at ultrasound; (b) including only women with short cervical length at ultrasound; and (c) including only women undergoing cerclage for dilated cervix.

First, we performed head-to-head meta-analyses and computed (a) summary risk ratios (RR) of the likelihood of detecting each categorical outcome in exposed versus unexposed women; and (b) summary mean differences (MDs) between exposed and unexposed women (for each continuous outcome), with their 95% confidence intervals (CIs). The relative intra-study heterogeneity was quantified using the I^2^ metric, and its 95% CIs were computed using the *heterogi* command in Stata. For categorical outcomes, data were combined using a random-effect generic inverse variance approach that enables the inclusion of diverse estimates of relative risk (i.e., OR and HR) into the same meta-analysis. From each paper, we extracted the adjusted estimates of each outcome, or, when these were not available, the unadjusted estimates. If a paper reported the results of different multivariate models, the most stringently controlled estimates (those from the model adjusting for more factors) were extracted. If different models controlled for the same number of covariates, the model containing the most relevant covariates was used for the analysis. In case different measures of risk were to be included in the same pooled analysis (e.g., OR and RR), the OR was converted into RR [29,30]. Furthermore, we stratified all analyses according to the study design (randomized controlled trial or observational).

Finally, in order to provide some estimates of the crude rates of each categorical outcome, we also performed meta-analysis of proportions, combining the data of women undergoing and not undergoing cerclage separately [29,30]. To account for between-study heterogeneity, the analyses were performed using a random-effect model.

Potential small study effect was assessed graphically, using funnel plots (displaying the ORs from individual comparisons versus their precision [1/SE]), and formally, using Egger’s regression asymmetry test [31]. All analyses were carried out using RevMan 5.4 (The Cochrane Collaboration, 2020) [32] and Stata, version 13.1 (Stata Corp., College Station, TX, 2013).

## Results

### Study selection and characteristics

**Fig 1:**
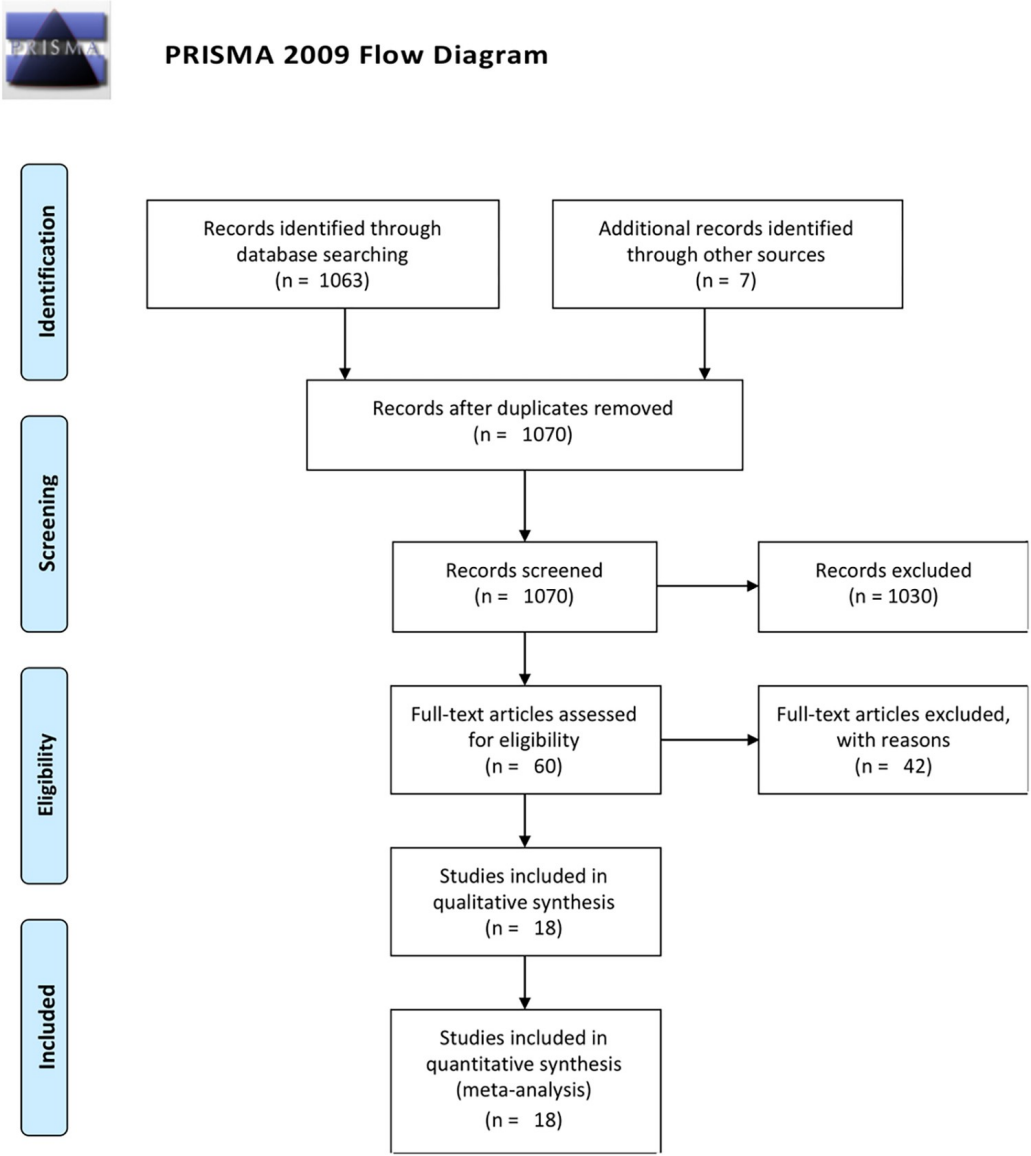
Systematic review flowchart.

A total of 1,070 studies were identified, 60 were assessed with respect to their eligibility for inclusion, and 18 included in the systematic review (Table 1, Fig 1) [15,17,20,33–47]. A list of the excluded studies and reasons for their exclusion is provided in S3 Table. These 18 studies included (after removing studies that included overlapping cases) 1,465 twin pregnancies with either short cervical length on ultrasound or cervical dilatation at physical examination. Four studies were randomized and 15 studies were observational.

**Table 1:** General characteristics of the studies included in the systematic review.

Author	Year	Country	Study design	Period considered	Inclusion criteria	Type of cerclage	Gestational age at cerclage placement (weeks)	Adjusted analysis*	Primary outcome	Twin pregnancies (*n*)
Qiu [33]	2023	China	Observational	2015–2021	Twin pregnancies with cervical dilatation (1 cm) at 18–26 weeks	McDonald	22, 8^Ξ^	Yes^a^	Gestational age at birth	99
Qiu [34]	2022	China	Observational	2015–2021	Twin pregnancies with short CL ≤25 mm at 18–26 weeks	McDonald	22, 9 ± 1.7^§^	Yes^a^	Gestational age at birth	90
Yao [20]	2022	China	Observational	2014–2020	Twin pregnancies with short CL ≤25 mm at 16–28 weeks	McDonald	16–28^ç^	Yes^b^	PTB <34 weeks	320
Zeng [35]	2022	China	Observational	2015–2020	Twin pregnancies with cervical dilatation and prolapsed membranes	McDonald	16+0–26+6^ç^	No	PTB <28 weeks	97
Pan [36]	2020	China	Observational	2015–2019	Twin pregnancies with asymptomatic cervical shortening or dilation at ultrasound and/or physical examination in mid-gestation	McDonald	23.7 (14.14–25.86)^Ψ^	Yes^c^	Gestational age at birth	62
Wu [17]	2020	Taiwan	Observational	2000–2017	DCDA twin pregnancies with a short cervical length (25 mm]	McDonald	NR^1^	No	Gestational age at birth	46
Roman [15]	2020	US	RCT	2015–2019	asymptomatic cervical dilation from 1–5 cm between 16 0/7 to 23 6/7 weeks	McDonald	20, 7 ± 1, 7^§^	Yes^b^	PTB <34 weeks	30
Han [37]	2020	US	Observational	2003–2016	Twin pregnancies with history of prior preterm birth, ultrasound-identified short cervix ≤2.5 cm, and cervical dilation ≥1.0 cm at 14–26 weeks	Shirodkar	20 (12–27)^Ψ^	Yes^d^	PTB <32 weeks	135
Qureshey [38]	2022	US	Observational	2006–2016	Twin pregnancies with short CL ≤25 mm at 15–24	McDonald	15–24^ç^	Yes^b^	Gestational age at birth	64
Abbasi [33]	2018	Canada	Observational	2003–2014	Dilated cervix and intact membranes before 25–week gestation	McDonald	21.5 ± 2.6^§^	No	PTB <34 weeks	36
Adams [40]	2018	US	Observational	2008–2014	Twin gestations identified with cervical length of ≤2.5 cm before 24 weeks gestation	McDonald	20.8 ± 1.9^§^	Yes^e^	PTB <35 weeks	82
Houlihan [41]	2016	UK	Observational	2006–2014	DC twin pregnancies with an ultrasound-determined cervical length of 1–24 mm at 16–24 weeks	McDonald	NR^1^	Yes^f^	PTB <32 weeks	80
Roman [42]	2016	US	Observational	1997–2014	Twin pregnancies identified with cervical dilation of >1 cm at 16–24 weeks	McDonald	20, 7 ± 1, 6^§^	Yes^g^	PTB <34 weeks	76
Roman [43]	2015	US	Observational	1995–2012	Asymptomatic twin pregnancies with TVU CL 25 mm at 16–24 weeks	Shirodkar or McDonald	NR^1^	Yes^h^	PTB <34 weeks	140
Roman [44]	2005	US	Observational	1996–2002	ALl twin pregnancies with CL ≤25 mm before 24 weeks	Shirodkar	20.8 (15.7–23.6)^Ψ^	No	PTB <32 weeks	31
Newman [45]	2002	US	Observational	1994–2001	Twin pregnancies with short CL ≤25 mm at 18–26	McDonald	18–26^ç^	No	PTB	33
Althuisius [46]	2001	The Netherland-Australia	RCT	1995–2000	Twin pregnancies with short CL (≤25 mm)	McDonald	Before 27 weeks	No	PTB <34 weeks	17
Rust [47]	2000	US	RCT	1998–1999	Twin pregnancies with short CL (≤25 mm)	McDonald	16–24^ç^	No	Gestational age at birth	27

^1^Detailed inclusion criteria not specified.

*Adjusted analyses referred to whether the computation of the risk analyses for the outcomes observed in the present systematic review were adjusted for any factor potentially associated with PTB.

^a^Analysis adjusted for maternal age, pregestational BMI, IVF, operative hysteroscopy, previous cervical surgery, previous spontaneous preterm birth, white blood count, C-reactive protein, neutrophil to lymphocyte ratio and the shortest cervical length at ultrasound.

^b^Not specified on which confounders the analyses were adjusted.

^c^Analyses adjusted for indomethacin, vaginal progesterone, antibiotics and basic demographic characteristics.

^d^Analyses adjusted for cerclage indication, clinical history, age, chorionicity, insurance type, race, BMI, IVF, and multifetal reduction.

^e^Analyses adjusted for age, BMI, race, vaginal progesterone use, and gestational age at shortest documented cervical length.

^f^Analyses adjusted for maternal age, BMI, racial origin, cigarette smoking, IVF, parity, and prior preterm delivery.

^g^Analyses adjusted for amniocentesis and vaginal progesterone administration.

^h^Analyses adjusted for gestational age at presentation and short cervical length.

^§^Standard deviation.

^Ψ^Median and interquartile.

^ç^Range.

^Ξ^Mean.

BMI, body mass index; CL, cervical length; DC, dichorionic; DCDA, dichorionic diamniotic; IVF, in vitro fertilization; NR, not reported; PTB, preterm birth; RCT, randomized controlled trial; TVU, transvaginal ultrasound.

The results of the quality assessment of the included studies using RoB2 tool are presented in Table 2. The study by Roman and colleagues was at low risk of bias, while those by Rust and colleagues and Althuisius and colleagues were at high risk of bias (Table 2).

**Table 2:** Risk of bias assessed using the Revised Cochrane risk-of-bias tool for randomized trials “RoB 2”.

Study ID	Randomization process	Deviations from intended interventions	Missing outcome data	Measurement of the outcome	Selection of the reported result	Overall bias
Roman and colleagues (2020)	Low risk	Low risk	Low risk	Low risk	Low risk	Low risk
Althuisius and colleagues (2001)	Low risk	High risk	Low risk	High risk	High risk	High risk
Rust and colleagues (2000)	Low risk	High risk	Low risk	High risk	High risk	High risk

According to this tool, the risk of bias of each included study is judged according to 5 domains: bias arising from the randomization process, bias due to deviations from intended interventions, bias due to missing outcome data, bias in the measurement of the outcome, and bias in selection of the reported result. Although the RoB2 tool does not provide an overall risk of bias assessment, the overall risk of bias was considered low if 4 or more domains were rated as low risk “not counting ‘other biases,’” with at least 1 being sequence generation or allocation concealment, according to what is reported in previous systematic reviews of intervention.

The results of the quality assessment of the observational studies are reported in Table 3. Most of the studies were of good quality; the main limitations of the included studies were small sample size, observational design, lack of subgroup analyses according to indication for cerclage, and heterogeneity in the outcomes observed and prenatal management of twin pregnancies undergoing cervical cerclage.

**Table 3:** Quality assessment of the included studies according to the NOS for cohort studies; a study can be awarded a maximum of one star for each numbered item within the Selection and Outcome categories. A maximum of 2 stars can be given for Comparability*.

Author	Year	Selection	Comparability	Outcome
Qiu	2023	★★★	★★	★★
Qiu	2022	★★	★	★
Yao	2022	★★★	★★	★★
Zeng	2022	★★	★★	★★
Pan	2020	★	★	★
Wu	2020	★★★	★★	★★
Han	2020	★★	★★	★★
Qureshey	2019	★★	★★	★★
Abbasi	2018	★★	★	★
Adams	2018	★★	★★	★
Houlihan	2016	★★	★★	★★
Roman	2016	★★	★★	★★
Roman	2015	★★★	★★	★★
Roman	2005	★★★	★★	★★
Newman	2002	★★	★★	★★

*Higher number of stars indicated a better quality of the study.

NOS, Newcastle–Ottawa scale.

Table 4 reports the main maternal and pregnancy characteristics potentially affecting the risk of PTB in twin pregnancies. There was no significant difference in the mean cervical length at ultrasound [*p* = 0.08] or cervical dilatation at physical examination (*p* = 0.05) between women receiving compared to those not receiving cervical cerclage. Likewise, there was no difference in the mean maternal age (*p* = 0.2), BMI (*p* = 0.3), nulliparity (*p* = 0.6), prior PTB (*n* = 0.7), and pharmacological intervention for reducing the risk of PTB, including indomethacin (*p* = 0.11), antibiotics (*p* = 0.4), and tocolytic drugs (*p* = 0.2) between the 2 groups. Women receiving cerclage were more likely to carry dichorionic gestations (RR: 0.63, 95% CI [0.44, 0.90], *p* = 0.01; 70/691 versus 84/556) and were diagnosed with short cervical length or cervical dilatation at earlier gestational ages compared to those not receiving cerclage (MD: −0.83 weeks, 95% CI [−1.47, −0.19], *p* = 0.01) (Table 4).

**Table 4:** Results of the meta-analyses comparing the likelihood of several baseline characteristics [or the mean age] between women undergoing cerclage versus women not undergoing cerclage.

Baseline characteristics	Number of studies	n/N vs. n/N	RR [95% CI]	*p* Value	I^2^ [95% CI], %
Monochorionic twins	11	70/691 vs. 84/556	0.63 [0.44, 0.90]	0.01	16 [0, 56]
Nulliparity	13	539/710 vs. 416/565	1.09 [0.82, 1.45]	0.6	31 [0, 64]
Prior preterm birth	13	61/566 vs. 61/471	0.94 [0.64, 1.38]	0.7	0 [0, 57]
In vitro fertilization	11	451/640 vs. 289/477	1.13 [0.95, 1.34]	0.2	65 [34, 82]
Progesterone use	7	242/445 vs. 173/306	1.00 [0.99, 1.01]	0.9	0 [0, 71]
Indomethacin use	5	148/183 vs. 96/174	1.65 [0.90, 3.02]	0.11	99 [99, 100]
Antibiotics use	8	245/370 vs. 220/322	0.96 [0.88, 1.06]	0.4	86 [74, 92]
Steroids use	6	191/267 vs. 104/169	1.05 [0.79, 1.41]	0.7	80 [58, 81]
Tocolysis	8	245/370 vs. 250/322	0.87 [0.71, 1.06]	0.2	98 [97, 98]
		**N/N**	**MD [95% CI]**		
Gestational age at diagnosis [weeks]	15	774/627	−0.83 [−1.47, −0.19]	0.01	77 [62, 86]
Maternal age at baseline	13	753/604	2.70 [−1.41, 6.81]	0.2	98 [98, 99]
Maternal BMI at baseline	12	669/467	2.76 [−1.79, 7.31]	0.3	99 [98, 99]
Cervical length at baseline	12	620/519	−0.54 [−0.94, 0.14]	0.09	60 [24, 79]
Cervical dilatation at baseline	7	319/216	−0.58 [−1.16, 0.00]	0.05	91 [83, 95]

BMI, body mass index; CI, confidence interval; MD, mean difference; RR, risk ratio.

### Synthesis of the results

#### Women with short cervical length at ultrasound or cervical dilatation at physical examination

**Fig 2:**
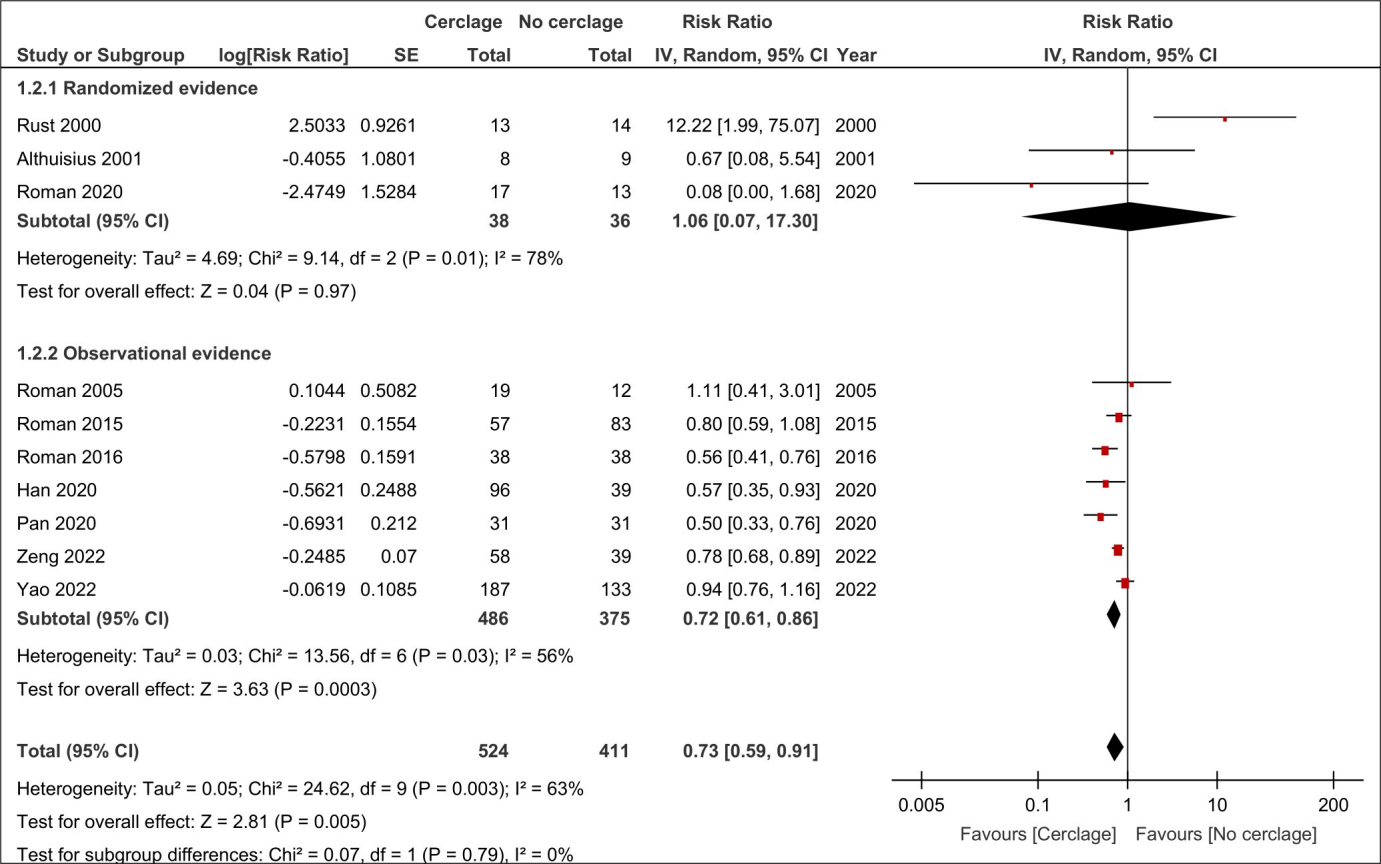
Pooled ORs for the risk of PTB <34 weeks of gestation in women with twin pregnancies undergoing compared to those not undergoing cervical cerclage. OR, odds ratio; PTB, preterm birth.

Placement of cervical cerclage in women with a twin pregnancy with a short cervix at ultrasound or cervical dilatation at physical examination was associated with a reduced risk of PTB <34 weeks of gestation (RR: 0.73, 95% CI [0.59, 0.91], *p* = 0.005, corresponding to a 16% difference in the absolute risk, AR) (Fig 2). The strength of such association was due to the reduced risk of PTB in women with cervical cerclage from the included observational studies (RR: 0.72, 95% CI [0.61, 0.86], *p* < 0.001), but not RCT (*p* = 0.9). Cervical cerclage was also associated with a reduced risk of PTB <32 (RR: 0.69, 95% CI [0.57, 0.84], *p* < 0.01, AR: 16.92%), <28 [RR: 0.54, 95% CI [0.43, 0.67], *p* < 0.01, AR: 18.29%) and <24 (RR: 0.48, 95% CI [0.23, 0.97], *p* = 0.04, AR: 15.57%) but not 37 weeks (*p* = 0.2) of gestation (Table 5). Likewise, cervical cerclage in twin pregnancies with either a short cervical length or cervical dilatation was associated with a prolonged gestational age at birth (MD: 2.32 weeks, 95% CI [0.99, 3.66, *p* < 0.001) and longer presentation to delivery interval (MD: 5.22 weeks, 95% CI [3.86, 6.59], *p* < 0.001) (Table 6).

**Table 5:** Women with a reduced cervical length on ultrasound and/or cervical dilatation at examination: Results of the head-to-head meta-analyses comparing the risk of selected categorical outcomes in women with twin pregnancies undergoing cerclage versus no cerclage.

*Outcomes [Ref*.*]*	Number of studies	Total women n/N vs. n/N	RR [95% CI]	*p* Value	I^2^ [95% CI], %
***Primary outcome*:**					
**Preterm birth <34th week**	**10**	**258/524 vs. 268/411**	**0.73 [0.59, 0.91]**	**0.005**	**63 [28, 81]**
- Randomized evidence	3	24/38 vs. 19/36	1.06 [0.07,17.3]	0.9	78 [29, 93]
- Observational evidence	7	234/486 vs. 249/375	0.72 [0.61, 0.86]	<0.001	56 [0, 81]
**Preterm birth <37th week**	**6**	**275/404 vs. 247/317**	**0.95 [0.87, 1.03]**	**0.2**	**3 [0, 75]**
- Randomized evidence	2	19/21 vs. 17/23	3.25 [0.57,18.7]	0.2	0 [––]
- Observational evidence	4	256/383 vs. 230/394	0.94 [0.87,1.02]	0.2	1 [0, 85]
**Preterm birth <32nd week**	**12**	**239/619 vs. 276/497**	**0.69 [0.57, 0.84]**	<0.001	**64 [27, 83]**
- Randomized evidence	3	19/38 vs. 16/36	1.28 [0.36, 4.54]	0.7	66 [0, 90]
- Observational evidence	9	220/581 vs. 260/461	0.68 [0.55, 0.82]	<0.001	64 [26, 82]
**Preterm birth <28th week**	**11**	**119/523 vs. 188/458**	**0.54 [0.43, 0.67]**	<0.001	**29 [0, 65]**
- Randomized evidence	3	12/38 vs. 12/36	1.35 [0.25, 7.14]	0.3	61 [0, 89]
- Observational evidence	8	107/485 vs. 176/422	0.52 [0.43, 0.64]	<0.001	19 [0, 62]
**Preterm birth <24th week**	**7**	**29/222 vs. 65/227**	**0.48 [0.23,0.97]**	**0.04**	**62 [14, 83]**
- Randomized evidence	3	7/38 vs. 11/36	0.77 [0.17,3.54]	0.7	45 [0, 84]
- Observational evidence	4	22/184 vs. 54/191	0.42 [0.116,1.11]	0.08	75 [30, 91]
**pPROM**	**8**	**105/404 vs. 126/324**	**0.75 [0.48, 1.16]**	**0.2**	**68 [32, 85]**
- Randomized evidence	3	16/38 vs. 9/36	1.57 [0.81, 3.04]	0.2	0 [0, 90]
- Observational evidence	5	89/366 vs. 117/288	0.60 [0.39, 0.92]	0.02	66 [12, 87]
**Chorioamnionitis**	**7**	**37/409 vs. 26/270**	**1.08 [0.54, 2.17]**	**0.8**	**50 [0, 79]**
- Randomized evidence	3	12/38 vs. 13/36	1.03 [0.23, 4.65]	0.9	70 [0, 91]
- Observational evidence	4	25/371 vs. 13/234	1.24 [0.70, 2.21]	0.5	0 [0, 85]
**Perinatal loss**	**9**	**131/980 vs. 223/676** *	**0.38 [0.25, 0.60]**	**<0.001**	**72 [44, 86]**
- Randomized evidence	3	9/76 vs. 22/54	0.43 [0.04, 4.63]	0.5	79 [32, 93]
- Observational evidence	6	122/904 vs. 201/622	0.42 [0.28, 0.63]	<0.001	71 [31, 87]
**Composite adverse outcome**	**8**	**418/904 vs. 283/488**	**0.69 [0.53, 0.90]**	**0.007**	**83 [69, 91]**
- Randomized evidence	1	14/30 vs. 3/6	0.93 [0.38, 2.27]	0.9	,,
- Observational evidence	7	404/874 vs. 280/482	0.67 [0.50, 0.90]	0.007	86 [73,93]
**5-min Apgar score <7**	**5**	**97/346 vs. 126/212**	**0.46 [0.29, 0.74]**	**0.001**	**75 [38,90]**
- Randomized evidence	1	9/34 vs. 22/26	0.31 [0.17, 0.56]	<0.001	--
- Observational evidence	4	88/312 vs. 104/186	0.50 [0.29, 0.89]	0.02	79 [42,92]
**RDS**	**4**	**70/224 vs. 56/160**	**1.13 [0.49, 2.62]**	**0.8**	**80 [47,92]**
- Randomized evidence	1	14/30 vs. 2/6	1.40 [0.42, 4.62]	0.6	--
- Observational evidence	3	56/194 vs. 54/154	1.09 [0.40, 2.97]	0.9	85 [54, 95]
**Sepsis**	**3**	**14/138 vs. 20/84**	**0.45 [0.24, 0.84]**	**0.01**	**0 [0, 90]**
- Randomized evidence	1	2/30 vs. 1/6	0.40 [0.04, 3.74]	0.4	--
- Observational evidence	2	12/108 vs. 19/78	0.46 [0.24, 0.87]	0.6	0 [––]
**Grade 3–4 IVH**	**4**	**16/224 vs. 42/160**	**0.32 [0.11, 0.92]**	**0.03**	**56 [0,85]**
- Randomized evidence	1	4/30 vs. 1/6	0.80 [0.11, 5.96]	0.8	--
- Observational evidence	3	12/194 vs. 41/154	0.26 [0.08, -0.90]	0.03	63 [0, 89]
**ROP**	**4**	**14/224 vs. 17/160**	**0.54 [0.10, 2.98]**	**0.2**	**66 [0, 88]**
- Randomized evidence	1	5/30 vs. 1/6	1.00 [0.10, 10.5]	0.99	--
- Observational evidence	3	9/194 vs. 16/154	0.46 [0.05, 4.31]	0.50	76 [20, 93]
**Birthweight <1,500 g**	**5**	**228/638 vs. 264/454**	**0.49 [0.33, 0.73]**	**<0.001**	**85 [66, 93]**
- Randomized evidence	1	21/34 vs. 24/26	0.13 [0.03, 0.67]	0.01	--
- Observational evidence	4	207/604 vs. 240/428	0.53 [0.36, 0.78]	0.001	87 [69, 95]
**NICU admission**	**7**	**452/822 vs. 306/464**	**0.75 [0.63, 0.90]**	**<0.001**	**74 [46, 88]**
- Randomized evidence	1	22/30 vs. 6/6	0.20 [0.01, 4.02]	0.3	--
- Observational evidence	6	430/792 vs. 300/458	0.76 [0.63, 0.91]	0.003	78 [51, 90]

*N. of fetuses.

RR, risk ratio; CI, confidence interval; n/N vs. n/N, number of women with the outcome/total number of women in the exposed [cerclage] and unexposed [no cerclage] group, respectively; PTB, preterm birth; pPROM, preterm premature rupture of membranes; RDS, respiratory distress syndrome; ROP, retinopathy of the prematurity; IVH, intraventricular hemorrhage; NICU, neonatal intensive care unit.

**Table 6:** Results of the meta-analyses comparing selected continuous perinatal outcomes in women with twin pregnancies undergoing cerclage versus no cerclage.

Outcomes	Number of studies [total sample]	MD [95% CI]	*p* Value	I^2^ [95% CI], %
***1*. *Cerclage for reduced cervical length on ultrasound or cervical dilatation at physical examination***				
**Gestational age at birth, (weeks)**	**17 [1,426]**	**2.32 [0.99, 3.66]**	**<0.001**	**86 [78, 90]**
- Randomized evidence	2 [48]	−0.09 [−0.26, 2.07]	0.9	[––]
- Observational evidence	15 [1,378]	2.54 [1.13, 3.95]	<0.001	87 [80, 91]
**Presentation to delivery interval, (weeks)**	**11 [801]**	**5.22 [3.86, 6.59]**	**<0.001**	**90 [84, 94]**
- Randomized evidence	1 [30]	5.40 [2.20, 8.60]	0.001	--
- Observational evidence	10 [771]	5.21 [3.77, 6.65]	<0.001	91 [86, 94]
**Birthweight, grams**	**15 [1,483]**	**300 [167, 433]**	**<0.001**	**90 [85, 93]**
- Randomized evidence	1 [60]	268 [132, 403]	<0.001	--
- Observational evidence	14 [1,423]	805 [467, 1143]	<0.001	90 [84, 93]
**NICU length of stay, [days]**	**6 [702]**	−**22.4 [**−**40.1,** −**4.7]**	**0.01**	**93 [87 96]**
- Randomized evidence	1 [56]	−22.2 [−41.4, −3.0]	0.02	--
- Observational evidence	5 [646]	−24.1 [−55.2, 7.0]	0.13	94 [89, 97]
***2*. *Cerclage for cervical dilatation at physical examination*:**				
**Gestational age at birth, (weeks)**	**5 [345]**	**3.64 [1.85, 5.43]**	**<0.001**	**68 [17, 88]**
- Randomized evidence	1 [30]	0.0 [−8.54, 8.54]	0.99	--
- Observational evidence	4 [314]	3.79 [1.92, 5.65]	<0.001	75 [30, 91]
**Presentation to delivery interval, (weeks)**	**5 [334]**	**5.43 [3.28, 7.57]**	**<0.001**	**95 [92, 97]**
- Randomized evidence	1 [30]	5.40 [2.20, 8.60]	0.01	--
- Observational evidence	4 [304]	5.43; [3.04, 7.81]	<0.001	97 [94, 98]
**Birthweight, grams**	**5 [375]**	**500 [297, 703]**	**<0.001**	**79 [51, 91]**
- Randomized evidence	1 [60]	805 [468, 1143]	<0.001	--
- Observational evidence	4 [315]	442 [230, 654]	<0.001	80 [46, 92]
**NICU length of stay, (days)**	**3 [203]**	−**36.7 [**−**56.4,** −**17.0]**	**<0.001**	**64 [0, 90]**
- Randomized evidence	1 [56]	−24.1 [−55.2, 7.0]	<0.001	--
- Observational evidence	2 [147]	−40.8 [−67.2, −14.4]	<0.001	79 [––]
***3*. *Cerclage for reduced cervical length on ultrasound [<25mm]*:**				
**Gestational age at birth, (weeks)**	**10 [989]**	**1.02 [**−**0.43, 2.46]**	**0.2**	**79 [62, 88]**
- Randomized evidence	1 [17]	−0.10 [−2.34, 2.14]	0.2	--
- Observational evidence	9 [972]	1.16 [−0.44, 2.76]	0.9	81 [65, 90]
**Presentation to delivery interval, (weeks)**				
- Observational evidence only	5 [346]	5.20 [2.29, 8.11]	<0.001	88 [74, 94]
**Birthweight, grams**				
- Observational evidence only	8 [911]	183 [−9.9, 376]	0.06	836 [68, 91]
***4*. *Cerclage for reduced cervical length on ultrasound [stratified by cervical length]*:**				
**Gestational age at birth, (weeks)- <15 mm**				
- Observational evidence only	5 [366]	2.34 [1.40, 3.28]	<0.001	0 [0, 79]
**Gestational age at birth, (weeks)- 15–25 mm**				
- Observational evidence only	4 [278]	1.36 [−1.26, 3.97]	0.3	57 [0, 89]
**Presentation to delivery interval, (weeks)—<15 mm**				
- Observational evidence only	4 [195]	3.79 [2.42, 5.15]	<0.001	0 [0, 85]
**Presentation to delivery interval, (weeks)- 15–25 mm**				
- Observational evidence only	3 [123]	3.00 [0.91, 5.08]	0.05	25 [0, 92]
**Birthweight, grams- *<*15 mm**				
- Observational evidence only	3 [544]	627 [57.6, 1,196]	0.003	98 [96, 99]
**Birthweight, grams- 15–25 mm**				
- Observational evidence only	2 [382]	78.1 [−3.76, 533]	0.7	85 [––]

CI, confidence interval; MD, mean difference; NICU, neonatal intensive care unit.

Conversely, there was no significant difference in the risk of pPROM (*p* = 0.2) or chorioamnionitis (*p* = 0.8) between women receiving and those not receiving cerclage. Pooled proportions for each of the explored outcomes in women with twin pregnancies receiving compared to those not receiving cerclage are reported in S4 Table.

Cerclage in twin pregnancy with short cervical length or cervical dilatation was also associated with a reduced risk of perinatal loss (RR: 0.38, 95% CI [0.25, 0.60], *p* < 0.001 AR: 19.62%), composite adverse outcome (RR: 0.69, 95% CI [0.53, 0.90], *p* = 0.007; AR: 11.75%), 5-min Apgar score <7 (RR: 0.46, 95% CI [0.29, 0.74], *p* = 0.00), neonatal sepsis (RR: 0.45, 95% CI [0.24, 0.84], *p* = 0.01), grade III or IV IVH (RR: 0.32, 95% [CI 0.11, 0.92], *p* = 0.03), birthweight <1,500 grams (RR: 0.49, 95% CI [0.33, 0.73], *p* < 0.01), and NICU admission (RR: 0.75, 95% CI [0.63, 0.90], *p* < 0.001] but not of RDS (*p* = 0.8) or ROP (*p* = 0.2).

Mean birthweight was also greater in twin pregnancies receiving cerclage [MD: 300 grams, 95% CI 167, 433; *p* < 0.01], while the length of stay in NICU was shorter [MD: −22.4 days, 95% CI −40.1, −4.7; *p* = 0.01]. When assessing the contribution of the different types of studies included in the reported results, the association between cervical cerclage and adverse maternal or perinatal outcome was exclusively due to the inclusion of observational studies but not RCTs.

Subgroup analyses according to the specific indication for cerclage (short cervical length at ultrasound or cervical dilation at physical examination) are presented in Tables 6–8.

In women with a CL ≤15 mm, placement of a cervical cerclage was associated with a reduced risk of PTB <34 weeks (RR: 0.74, 95% CI [0.58, 0.95], *p* < 0.001, AR: 29.17%) and composite adverse neonatal outcome (RR: 0.75, 95% CI [0.61, 0.92; 0.03], *p* = 0.003, AR: 22.64%) (Table 7). Cerclage was also associated with a later gestational age at birth (MD: 2.34, 95% CI 1.40, 3.28, *p* < 0.001) and a longer presentation to delivery interval (MD: 3.79, 95% CI [2.42, 5.15], *p* < 0.001) and neonatal birthweight (MD: 627 grams, 95% CI [57.6, 1,196], *p* = 0.003). The association between cerclage and reduced risk of maternal and perinatal outcome was due to the inclusion of observational studies, while the RCT did not show any potential beneficial effect of cerclage in affecting such outcomes.

**Table 7:** Women with a reduced cervical length on ultrasound: Results of the head-to-head meta-analyses comparing the risk of selected categorical outcomes in women with twin pregnancies undergoing cerclage versus no cerclage.

*Outcomes*	Number of studies	Total women n/N vs. n/N	RR [95% CI]	*p* Value	I^2^ [95% CI], %
***Primary outcome*:**					
**Preterm birth <34th week**	**5**	**143/248 vs. 135/251**	**0.99 [0.68, 1.45]**	**0.9**	**55 [0, 83]**
- Randomized evidence	2	12/21 vs. 6/23	3.01 [0.17, 51.9]	0.5	0 [––]
- Observational evidence	3	131/263 vs. 129/228	0.90 [0.76, 1.07]	0.2	0 [0, 90]
***By cervical length*:**					
**Preterm birth <34th week—<15mm**					
- Observational evidence only	2	29/56 vs. 51/63	0.74 [0.58, 0.95]	0.02	0 [––]
**Preterm birth <34th week—15–25mm**					
- Observational evidence only	1	11/21 vs. 17/21	0.65 [0.41, 1.02]	0.07	--
**Preterm birth <37th week**	**5**	**237/308 vs. 223/278**	**0.96 [0.88, 1.04]**	**0.3**	**0 [0, 79]**
- Randomized evidence	2	19/21 vs. 17/23	3.25 [0.57, 18.7]	0.2	0 [––]
- Observational evidence	3	218/287 206/255	0.96 [0.88, 1.04]	0.2	0 [0, 90]
**Preterm birth <32nd week**	**6**	**110/327 vs. 108/290**	**0.90 [0.71, 1.13]**	**0.9**	**5 [0, 76]**
- Randomized evidence	2	8/21 vs. 3/23	2.89 [0.86, 9.78]	0.09	0 [––]
- Observational evidence	4	102/306 vs. 105/267	0.86 [0.70, 1.07]	0.2	5 [0, 76]
**Preterm birth <28th week**	**6**	**46/327 vs. 55/290**	**0.75 [0.53, 1.08]**	**0.13**	**0 [0, 75]**
- Randomized evidence	2	5/21 vs. 1/23	3.98 [0.72, 22.0]	0.11	0 [––]
- Observational evidence	4	41/306 vs. 54/267	0.70 [0.48, 1.01]	0.06	0 [0, 85]
**Preterm birth <24th week**	**3**	**9/78 vs. 5/106**	**2.08 [0.80, 5.39]**	**0.13**	**0 [0, 90]**
- Randomized evidence	2	2/21 vs. 0/23	2.23 [0.32, 15.7]	0.4	0 [––]
- Observational evidence	1	7/57 vs. 5/83	2.03 [0.68, 6.06]	0.2	--
**pPROM**	**3**	**46/208 vs. 47/156**	**0.76 [0.44, 1.32]**	**0.3**	**12 [0, 91]**
- Randomized evidence	2	5/21 vs. 4/23	1.36 [0.31, 6.07]	0.7	0 [––]
- Observational evidence	1	41/187 vs. 43/133	0.68 [0.47, 0.98]	0.04	--
**Chorioamnionitis**	**3**	**8/208 vs. 3/156**	**2.29 [0.76, 6.93]**	**0.14**	**0 [0, 90]**
- Randomized evidence	2	6/21 vs. 2/23	2.54 [0.74, 8.79]	0.7	0 [––]
- Observational evidence	1	2/187 vs. 1/133	1.52 [0.13, 17.8]	0.14	--
**Perinatal loss**	**4**	**61/502 vs. 60/372** *	**0.77 [0.55, 1.07]**	**0.12**	**0 [0, 85]**
- Randomized evidence	2	3/42 vs. 2/28	0.50 [0.31, 7.20]	0.6	0 [––]
- Observational evidence	2	58/460 vs. 58/344	0.74 [0.52, 1.05]	0.09	1 [––]
**Composite adverse outcome**	**4**	**185/449 vs. 144/340**	**1.11 [0.63, 1.96]**	**0.7**	**68 [7, 89]**
- Randomized evidence	2	18/42 vs. 12/46	0.87 [0.74, 1.02]	0.06	87 [––]
- Observational evidence	2	167/407 vs. 132/294	1.65 [0.23, 11.8]	0.9	0 [––]
***By cervical length*:**					
**Composite adverse outcome—<15 mm**					
- Observational evidence only	2	54/104 vs. 85/114	0.75 [0.61, 0.92]	0.03	4 [––]
**Composite adverse outcome—15–25 mm**					
- Observational evidence only	1	14/41 vs. 22/30	0.47 [0.29, 0.75]	0.002	--
**RDS**	**3**	**29/128 vs. 15/122**	**2.32 [0.66, 8.10]**	**0.2**	**64 [0, 90]**
- Randomized evidence	2	14/86 vs. 12/76	1.03 [0.51, 2.08]	0.9	0 [––]
- Observational evidence	1	15/42 vs. 3/46	4.78 [1.65, 13.8]	0.004	--
**Sepsis**					
- Randomized evidence only	2	0/42 vs. 2/46	0.54 [0.07, 3.96]	0.5	0 [––]
**Grades 3–4 IVH**	**3**	**4/128 vs. 5/122**	**0.85 [0.25, 2.88]**	**0.8**	**0 [0, 90]**
- Randomized evidence	2	1/42 vs. 3/46	0.56 [0.10, 3.06]	0.5	0 [––]
- Observational evidence	1	3/86 vs. 2/76	1.33 [0.23, 7.69]	0.8	--
**Birthweight <1,500 g—all studies**	**3**	**139/416 vs. 105/312**	**1.53 [0.51, 4.59]**	**0.5**	**81 [41, 94]**
- Randomized evidence	2	19/42 vs. 7/46	2.73 [1.00, 7.42]	0.05	23 [––]
- Observational evidence	1	120/374 vs. 98/266	0.87 [0.70, 1.08]	0.2	--
**NICU admission—all studies**	**3**	**2036/423 vs. 170/312**	**0.90 [0.77, 1.07]**	**0.2**	**12 [0, 91]**
- Randomized evidence	1	5/16 vs. 9/18	0.63 [0.26, 1.48]	0.3	--
- Observational evidence	2	201/407 vs.161/294	0.92 [0.76, 1.12]	0.4	37 [––]

*N. of fetuses.

RR, risk ratio; CI, confidence interval; n/N vs. n/N, number of women with the outcome/total number of women in the exposed [cerclage] and unexposed [no cerclage] group, respectively; pPROM, preterm premature rupture of membranes; IVH, intraventricular hemorrhage; NICU, neonatal intensive care unit; RDS, respiratory distress syndrome.

Conversely, cerclage in women with a cervical length between 15 and 25 mm was not associated with a reduced risk of any of the main ofutcomes assessed in this systematic review.

In women with twin pregnancy and cervical dilatation at physical examination, placement of a cervical cerclage was associated with a reduced risk of PTB <34 (RR: 0.68, 95% CI [0.57, 0.80], *p* = 0.001), <32 (RR: 0.59, 95% CI [0.50, 0.70], *p* = 0.001), <28 (RR: 0,47 95% CI [0.36, 0.62], *p* < 0.001), and <24 weeks (RR: 0.32 95% CI [0.21, 0.48], *p* < 0.001), but not that of pPROM (*p* = 0.3), chorioamnionitis (*p* = 0.5). Cerclage in these women also reduced the risk of perinatal loss (RR: 0.30, 95% CI [0.16, 0.55], *p* < 0.001), Apgar score <7 at 5 min (RR: 0.49, 95% CI [0.27, 0.90], *p* < 0.001), birthweight <1,500 grams (RR: 0.41, 95% CI [0.31, 0.55], *p* < 0.001), and admission to NICU (RR: 0.67, 95% CI [0.52, 0.88], *p* = 0.003), but not that of RDS (*p* = 0.5), grades III and IV IVH (*p* = 0.2) or ROP (*p* = 0.3) (Table 8). Such association was due to the inclusion of observational studies but no RCTs. Unfortunately, we could not perform meaningful pooled subgroup analyses according to different degrees of cervical dilatation [>2, >3, >4 cm]. Likewise, we could not perform sub-analyses according to chorionicity.

**Table 8:** Women with cervical dilatation at physical examination: Results of the head-to-head meta-analyses comparing the risk of selected categorical outcomes in women with twin pregnancies undergoing cerclage versus no cerclage.

*Outcomes*	Number of studies	Total women n/N vs. n/N	RR [95% CI]	*p* Value	I^2^ [95% CI], %
***Primary outcome*:**					
**Preterm birth <34th week**	**5**	**111/194 vs. 107/116**	**0.68 [0.57, 0.80]**	**<0.001**	**43 [0, 78]**
- Randomized evidence	1	12/17 vs. 13/13	0.08 [0.00, 1.68]	0.11	--
- Observational evidence	4	99/177 vs. 94/103	0.66 [0.53, 0.82]	0.002	60 [0, 84]
**Preterm birth <32nd week**	**6**	**130/246 vs. 147/163**	**0.59 [0.50, 0.70]**	**<0.001**	**19 [0, 70]**
- Randomized evidence	1	11/17 vs. 13/13	0.66 [0.46, 0.95]	0.03	--
- Observational evidence	5	119/229 vs. 130/154	0.61 [0.44, 0.83]	0.002	85 [59, 90]
**Preterm birth <28th week**	**5**	**82/192 vs. 126/146**	**0.47 [0.36, 0.62]**	**<0.001**	**39[0, 79]**
- Randomized evidence	1	7/17 vs. 11/13	0.49 [0.26, 0.90]	0.02	--
- Observational evidence	4	75/175 vs. 115/133	0.50 [0.39, 0.66]	<0.001	53 [0, 83]
**Preterm birth <24th week**	**4**	**25/140 vs. 56/99**	**0.32 [0.21, 0.48]**	**<0.001**	**0 [0, 68]**
- Randomized evidence	1	5/17 vs. 11/13	0.35 [0.16, 0.75]	0.007	--
- Observational evidence	3	20/123 vs. 45/86	0.31 [0.18, 0.42]	<0.001	16 [0, 77]
**pPROM**	**4**	**62/192 vs. 71/146**	**0.68 [0.33, 1.40]**	**0.3**	**80 [18, 91]**
- Randomized evidence	1	11/17 vs. 5/13	1.78 [0.68, 3.74]	0.2	--
- Observational evidence	4	51/175 vs. 66/133	0.62 [0.33, 1.14]	0.07	74 [0, 89]
**Chorioamnionitis**	**3**	**17/102 vs. 12/61**	**1.95 [0.32, 11.7]**	**0.5**	**93 [85, 97]**
- Randomized evidence	1	6/17 vs. 11/13	0.42 [0.21, 0.83]	<0.001	--
- Observational evidence	3	11/85 vs. 1/48	2.90 [0.56, 14.98]	0.203	0 [––]
Perinatal loss *	**3**	**59/226 vs. 129/180**	**0.30 [0.16, 0.55]**	**<0.001**	**77 [26, 93]**
- Randomized evidence	1	6/34 vs. 20/26	0.06 [0.02, 0.23]	<0.001	--
- Observational evidence	2	53/192 vs. 109/154	0.39 [0.28, 0.53]	<0.001	34 [––]
**Composite adverse outcome**	**4**	**114/258 vs**. **72/116**	**0.64 [0.39, 1.04]**	**0.07**	**79 [42, 92]**
- Randomized evidence	1	14/30 vs. 3/26	0.93 [0.38, 2.27]	0.9	--
- Observational evidence	3	100/228 vs. 69/90	0.59 [0.34, 1.03]	0.07	84 [52, 95]
**5-min Apgar score <7**	**4**	**84/280 vs. 89/150**	**0.49 [0.27, 0.90]**	**0.02**	**80 [46, 92]**
- Randomized evidence	1	9/30 vs. 22/26	0.31 [0.17, 0.56]	<0.001	--
- Observational evidence	3	75/250 vs. 67/124	0.59 [0.27, 1.21]	0.14	83 [50, 95]
**RDS**	**2**	**39/84 vs. 41/68**	**0.71 [0.27, 1.83]**	**0.5**	**63 [––]**
- Randomized evidence	1	14/30 vs. 2/26	0.40 [0.42, 4.62]	0.6	--
- Observational evidence	1	25/54 vs. 39/42	0.50 [0.37, 0.68]	<0.001	--
**Sepsis**	**2**	**9/84 vs. 11/68**	**0.52 [0.23, 1.15]**	**0.11**	**0 [––]**
- Randomized evidence	1	2/30 vs. 1/26	0.40 [0.04 3.74]	0.4	--
- Observational evidence	1	7/54 vs. 10/42	0.54 [0.23, 1.27]	0.2	--
**Grades 3–4 IVH**	**2**	**6/84 vs. 18/68**	**0.24 [0.03, 2.03]**	**0.2**	**66 [––]**
- Randomized evidence	1	4/30 vs. 1/26	0.80 [0.11, 5.96]	0.8	--
- Observational evidence	1	2/54 vs. 17/42	0.09 [0.02, 2.41]	0.002	--
**ROP**	**2**	**6/84 vs. 10/68**	**0.29 [0.03, 3.05]**	**0.3**	**53 [––]**
- Randomized evidence	1	5/30 vs. 1/26	1.00 [0.10, 10.5]	0.99	--
- Observational evidence	1	1/54 vs. 9/42	0.09 [0.01, 0.81]	0.03	--
**Birthweight <1,500 g**	**3**	**84/202 vs. 115/126**	**0.41 [0.31, 0.55]**	**<0.001**	**32 [0, 93]**
- Randomized evidence	1	31/34 vs. 24/26	0.13 [0.03, 0.67]	0.001	--
- Observational evidence	2	63/168 vs. 91/100	0.43 [0.35, 0.54]	<0.001	0 [––]
**NICU admission**	**3**	**117/176 vs. 71/92**	**0.67** [**0.52, 0.88]**	**0.003**	**63 [0, 89]**
- Randomized evidence	1	22/30 vs. 6/26	0.20 [0.01, 4.02]	0.3	--
- Observational evidence	2	95/168 vs. 65/100	0.68 [0.52, 0.89]	0.006	79 [––]

*N. of fetuses.

RR, risk ratio; CI, confidence interval; n/N vs. n/N, number of women with the outcome/total number of women in the exposed [cerclage] and unexposed [no cerclage] group, respectively; pPROM, preterm premature rupture of membranes; RDS, respiratory distress syndrome; ROP, retinopathy of the prematurity; IVH, intraventricular hemorrhage; NICU, neonatal intensive care unit.

### Grade

Assessment of the quality of retrieved evidence according to GRADE is presented in S5 Table. Overall, a low quality of evidence showed that cerclage can reduce the risk of PTB <34 weeks of gestation in women with a short cervix at ultrasound or cervical dilatation at physical examination and this could be due to the considerable inclusion of observational studies, indirectness of evidence, imprecision of results, and publication bias.

## Discussion

The findings from this systematic review showed that there is still a low grade of evidence that cerclage may prevent PTB in twin pregnancies. Although the placement of cervical cerclage in women with short cervical length <15 mm or cervical dilatation may be potentially associated with a reduced risk of PTB and adverse perinatal outcome compared with no intervention, this evidence is mainly supported by observational studies, but no RCTs, although only 1 trial was published in the last few years.

This is, to the best of our knowledge, the largest and most up-to-date systematic review and meta-analysis on the role of cervical cerclage in affecting PTB in twin pregnancies. Previous systematic reviews have addressed the association between cerclage and perinatal outcome in twins [12,48–51]. Compared to this review, the present study includes a well-defined population of twin pregnancies at high risk of PTB, defined as the presence of a short cervical length at ultrasound or cervical dilatation at physical examination, a large number of outcomes explored, stratification of the analyses according to cervical length at ultrasound or cervical dilatation, and computation of the observed outcome according to the study design (observational versus RCT).

The small number of cases in some of the included studies, their nonrandomized design, lack of standardized criteria for prenatal assessment, and management of twin pregnancies at higher risk of PTB represent the main limitation of the present review. The most significant limitation of the present systematic review relies on the inclusion of mainly observational studies. The large majority of RCTs were old, with a very small number of cases and a heterogeneous population of twin pregnancies, thus considerably limiting the robustness of their findings. Only 1 RCT was published in the recent past, showing a potential beneficial role of cerclage in women with cervical dilatation. However, even this trial, despite being powered for its primary outcome, was limited by a very small number of included cases and also by potential confounders such as the use of indomethacin and antibiotics in the intervention arm. The assessment of the role of cerclage in twin pregnancies with different cut-offs of cervical length was limited by the small number of included cases and an even smaller number of events that might have precluded a robust assessment of the strength of association between cerclage placement and neonatal morbidity in twins.

PTB is the leading cause of perinatal mortality and morbidity worldwide with an estimated societal economic burden in the United States of $26.2 billion annually. Therefore, identifying pregnancies at higher risk of PTB is pivotal in applying preventive strategies. In singleton pregnancies, assessment of cervical length at mid-gestation allows the identification of women with a higher likelihood of delivering preterm. Several preventive strategies for PTB in singleton pregnancies have been proposed. A recent network meta-analysis comparing progesterone, pessary, or cerclage for the prevention of PTB in singleton pregnancies has reported that vaginal progesterone in pregnancies at high risk was the only intervention with consistent effectiveness and was associated with a significant reduction in the risk of PTB <34 and <37 weeks’ gestation and in the risk of neonatal death [10]. Placement of cervical cerclage is commonly considered a secondary preventive strategy for PTB, especially in asymptomatic women with reduced cervical length already taking progesterone therapy. A recent individual patient data (IPD) meta-analysis comparing insertion of cerclage with expectant management reported no significant reduction in PTB <35 weeks’ gestation in asymptomatic women with a singleton pregnancy and a short second trimester cervical length (<25 mm). However, a subgroup analysis of the same cohort including women with cervical length <10 mm demonstrated a significant reduction in PTB <35 weeks [52]. On this basis, most relevant national and international societies suggest follow-up ultrasound scans every 1 to 2 weeks up to 24 weeks’ gestation in women with reduced cervical length and recommend cerclage placement in those whose cervix shortens to <10 mm despite using progesterone [53].

Screening for PTB in twin pregnancies is more controversial. The International Society of Ultrasound in Obstetrics and Gynecology (ISUOG) recommends that cervical length should be assessed in both monochorionic and dichorionic twin pregnancies at 20 weeks of gestation [54]. However, although in asymptomatic women with twin pregnancies, a short cervical length at ultrasound is associated with a higher risk of PTB, the diagnostic performance of this test is lower than in singletons [55,56]. Furthermore, the optimal cut-off of cervical length to define a twin pregnancy at increased risk of PTB remains controversial. Conventionally, a cut-off of 25 mm, as in singletons, is used.

The effectiveness of the most common strategies for the prevention of PTB is also controversial. Bed rest, progesterone therapy, Arabin cervical pessary, or oral tocolytics do not reduce the risk of PTB in twin pregnancies. A recent network meta-analysis reported that cervical pessary, progesterone, and cerclage do not show a significant effect in reducing the rate of PTB or perinatal morbidity in twins, either in an unselected population of twins or in pregnancies with a short cervix [12]. However, in this review, only 3 small RCTs on cerclage in twins were included. These studies were published almost 2 decades ago and were limited by the very small number of included cases and an even smaller number of events, as well as large heterogeneity in the prenatal management of twin pregnancies with risk factors for PTB, thus preventing robust conclusions on the lack of effectiveness of cerclage in twin pregnancies. More recently, an RCT by Roman and colleagues has reported that, in asymptomatic twin pregnancies with cervical dilation of 1 to 5 cm between 16^+0^ and 23^+6^ weeks of gestation, placement of cervical cerclage was associated with a significant reduction of PTB <34, 32, 28, and 24 weeks of gestation and a higher mean gestational age at birth (29.05 ± 1.7 versus 22.5 ± 3.9 weeks). Perinatal mortality was also significantly reduced in the cerclage group compared with the no cerclage group [15]. Since the publication of this trial, many observational studies on the role of cerclage in twin pregnancies have been published, challenging the old dogma of its lack of effectiveness in preventing PTB.

In the current review, we have also confirmed the potential beneficial role of cerclage in reducing the risk of PTB and neonatal morbidity in twin pregnancies with a cervical length <15 mm, similar to that reported in singleton pregnancies. Conversely, in women with a cervical length of 15 to 25 mm, cerclage was not associated with a reduction in the risk of any of the outcomes assessed. These findings are consistent with those of studies on the predictive accuracy of ultrasound in twin pregnancies that report that lower cut-offs of cervical length compared to those used in singletons better predict PTB in multiple gestations [57]. Mid-trimester mean cervical length is less in twin compared to singleton gestations and it is biologically plausible that this reduction may be due to the effect of uterine overdistension on the cervix, leading to a relative shortening compared to singletons, without being associated with an increased risk of PTB. On this basis, placement of cervical cerclage in women with cervical length >15 mm on ultrasound should not be recommended, although these findings are based on observational evidence.

Although the findings from this meta-analysis suggest a potential beneficial role of cervical cerclage in reducing the risk of PTB and improving neonatal outcome in women at risk, the inclusion of mainly observational studies significantly affect the robustness of the results and should be confirmed in adequately powered RCTs. Only 3 RCTs were included, with a very small number of women allocated to cerclage or standard care. Ideally, an RCT of the role of cerclage in twin pregnancies should include women with short cervix on ultrasound or cervical dilatation separately and be adequately powered to investigated maternal and neonatal outcomes. Furthermore, this trial should share an objective protocol of prenatal assessment of women at risk and management of women before and after cerclage placement, including the timing of ultrasound assessment of cervical and preventive strategies of PTB, including progesterone and tocolysis.

Twin pregnancies undergoing cerclage for short cervix at ultrasound or cervical dilatation at physical examination have a lower risk of PTB and perinatal mortality and morbidity compared to those not undergoing such intervention. However, these findings are driven mainly from observational studies, thus limiting the robustness of the results. The findings from the present systematic review highlight the need for designing an appropriately powered RCT to elucidate whether the placement of a cervical cerclage may prevent PTB in women presenting with short cervical length at ultrasound assessment or cervical dilatation at physical examination.

## Supporting information

S1 Table Search strategy. (DOCX)

S2 Table Prisma checklist. (DOCX)

S3 Table Excluded studies and reason for exclusion. (DOCX)

S4 Table Pooled proportions for the perinatal outcomes explored in the present systematic review (95% confidence intervals between parentheses) in twin compared pregnancies undergoing compared to those not undergoing cerclage. (DOCX)

S5 Table GRADE assessment of the primary outcome. (DOCX)

S1 Fig Funnel plot of the effect estimates vs. their standard errors (outcome: risk of preterm birth <34th week in women undergoing cerclage versus no cerclage—women with a reduced cervical length on ultrasound and/or cervical dilatation at examination). (DOCX)

S2 Fig Funnel plot of the effect estimates vs. their standard errors (outcome: risk of preterm birth <32nd week in women undergoing cerclage versus no cerclage—women with a reduced cervical length on ultrasound and/or cervical dilatation at examination). (DOCX)

S3 Fig Funnel plot of the effect estimates vs. their standard errors (outcome: risk of preterm birth <28th week in women undergoing cerclage versus no cerclage—women with a reduced cervical length on ultrasound and/or cervical dilatation at examination). (DOCX)

S4 Fig Funnel plot of the effect estimates vs. their standard errors (outcome: gestational age in women undergoing cerclage versus no cerclage—women with a reduced cervical length on ultrasound and/or cervical dilatation at examination). (DOCX)

S5 Fig Funnel plot of the effect estimates vs. their standard errors (outcome: gestational age in women undergoing cerclage versus no cerclage—women with a reduced cervical length on ultrasound). (DOCX)

S6 Fig Funnel plot of the effect estimates vs. their standard errors (outcome: presentation to delivery interval in women undergoing cerclage versus no cerclage—women with a reduced cervical length on ultrasound and/or cervical dilatation at examination). (DOCX)

S7 Fig Funnel plot of the effect estimates vs. their standard errors (outcome: birthweight in women undergoing cerclage versus no cerclage—women with a reduced cervical length on ultrasound and/or cervical dilatation at examination). (DOCX)

## Abbreviations

AR: absolute risk
CI: confidence interval
IPD: individual patient data
ISUOG: International Society of Ultrasound in Obstetrics and Gynecology
IVH: intraventricular hemorrhage
MC: monochorionic
MD: mean difference
MeSH: medical subject heading
NEC: necrotizing enterocolitis
NICU: neonatal intensive care unit
NOS: Newcastle–Ottawa scale
pPROM: preterm prelabor rupture of the membrane
PTB: preterm birth
RCT: randomized controlled trial
RDS: respiratory distress syndrome
ROP: retinopathy of prematurity
RR: risk ratio
sFGR: selective fetal growth restriction
TTTS: twin-to-twin transfusion syndrome

## References

[1] D’Antonio F, Khalil A, Dias T, Thilaganathan B, Bahamie A, Bhide A, et al. Early fetal loss in monochorionic and dichorionic twin pregnancies: analysis of the Southwest Thames Obstetric Research Collaborative (STORK) multiple pregnancy cohort. Ultrasound Obstet Gynecol. 2013;41(6):632–6. doi: 10.1002/uog.12363 23208731

[2] Buca D, Pagani G, Rizzo G, Familiari A, Flacco M, Manzoli L, et al. Outcome of monochorionic twin pregnancy with selective intrauterine growth restriction according to umbilical artery Doppler flow pattern of smaller twin: systematic review and meta-analysis. Ultrasound Obstet Gynecol. 2017;50(5):559–68. doi: 10.1002/uog.17362 27859836

[3] D’Antonio F, Odibo AO, Prefumo F, Khalil A, Buca D, Flacco ME, et al. Weight discordance and perinatal mortality in twin pregnancy: systematic review and meta-analysis. Ultrasound Obstet Gynecol. 2018;52(1):11–23. doi: 10.1002/uog.18966 29155475

[4] Townsend R D’Antonio F, Sileo FG, Kumbay H, Thilaganathan B, Khalil A. Perinatal outcome of monochorionic twin pregnancy complicated by selective fetal growth restriction according to management: systematic review and meta-analysis. Ultrasound Obstet Gynecol. 2019;53(1):36–46. doi: 10.1002/uog.20114 30207011

[5] Sileo F, Curado J, D’Antonio F, Benlioglu C, Khalil A. Incidence and outcome of prenatal brain abnormalities in twin-to-twin transfusion syndrome: systematic review and meta-analysis. Ultrasound Obstet Gynecol. 2022. 10.1002/uog.24895 35233861

[6] Di Mascio D, Khalil A, D’Amico A, Buca D, Benedetti Panici P, Flacco M, et al. Outcome of twin–twin transfusion syndrome according to Quintero stage of disease: systematic review and meta-analysis. Ultrasound Obstet Gynecol. 2020;56(6):811–20. doi: 10.1002/uog.22054 32330342

[7] Sileo FG D’antonio F, Benlioglu C, Bhide A, Khalil A. Perinatal outcomes of twin pregnancies complicated by late twin-twin transfusion syndrome: A systematic review and meta-analysis. Acta Obstet Gynecol Scand. 2021;100(5):832–42. doi: 10.1111/aogs.14066 33337543

[8] D’Antonio F, Benlioglu C, Sileo FG, Thilaganathan B, Papageorghiou A, Bhide A, et al. Perinatal outcomes of twin pregnancies affected by early twin-twin transfusion syndrome: A systematic review and meta-analysis. Acta Obstet Gynecol Scand. 2020;99(9):1121–34. doi: 10.1111/aogs.13840 32162305

[9] Litwinska E, Syngelaki A, Cimpoca B, Frei L, Nicolaides KH. Outcome of twin pregnancy with two live fetuses at 11–13 weeks’ gestation. Ultrasound Obstet Gynecol. 2020 Jan;55(1):32–38. doi: 10.1002/uog.21892 Epub 2019 Dec 13. . 31613412

[10] Jarde A, Lutsiv O, Beyene J, McDonald SD. Vaginal progesterone, oral progesterone, 17-OHPC, cerclage, and pessary for preventing preterm birth in at-risk singleton pregnancies: an updated systematic review and network meta-analysis. BJOG. 2019;126(5):556–67. doi: 10.1111/1471-0528.15566 30480871

[11] Rafael TJ, Berghella V, Alfirevic Z. Cervical stitch (cerclage) for preventing preterm birth in multiple pregnancy. Cochrane Database Syst Rev. 2014(9). doi: 10.1002/14651858.CD009166.pub2 25208049 PMC10629495

[12] D’Antonio F, Berghella V, Di Mascio D, Saccone G, Sileo F, Flacco ME, et al. Role of progesterone, cerclage and pessary in preventing preterm birth in twin pregnancies: A systematic review and network meta-analysis. Eur J Obstet Gynecol Reprod Biol. 2021;261:166–77. doi: 10.1016/j.ejogrb.2021.04.023 33946019

[13] Jarde A, Lutsiv O, Park CK, Barrett J, Beyene J, Saito S, et al. Preterm birth prevention in twin pregnancies with progesterone, pessary, or cerclage: a systematic review and meta-analysis. BJOG. 2017 Jul;124(8):1163–1173. doi: 10.1111/1471-0528.14513 Epub 2017 Feb 8. . 28176485

[14] Conde-Agudelo A, Romero R, Nicolaides K, Chaiworapongsa T, O’Brien JM, Cetingoz E, et al. Vaginal progesterone vs cervical cerclage for the prevention of preterm birth in women with a sonographic short cervix, previous preterm birth, and singleton gestation: a systematic review and indirect comparison metaanalysis. Am J Obstet Gynecol. 2013;208(1):42.e1–42.e18. 10.1016/j.ajog.2012.10.877 PMC3529767 23157855

[15] Roman A, Zork N, Haeri S, Schoen CN, Saccone G, Colihan S, et al. Physical examination–indicated cerclage in twin pregnancy: a randomized controlled trial. Am J Obstet Gynecol. 2020;223(6):902.e1–902.e11. doi: 10.1016/j.ajog.2020.06.047 32592693

[16] Freegard GD, Donadono V, Impey LW. Emergency cervical cerclage in twin and singleton pregnancies with 0-mm cervical length or prolapsed membranes. Acta Obstet Gynecol Scand. 2021;100(11):2003–8. doi: 10.1111/aogs.14255 34476806

[17] Wu F-T, Chen Y-Y, Chen C-P, Sun F-J, Chen C-Y. Outcomes of ultrasound-indicated cerclage in twin pregnancies with a short cervical length. Taiwan J Obstet Gynecol. 2020;59(4):508–13. 32653121 10.1016/j.tjog.2020.05.007

[18] Rottenstreich A, Levin G, Kleinstern G, Zigron R, Rottenstreich M, Elchalal U, et al. History-indicated cervical cerclage in management of twin pregnancy. Ultrasound Obstet Gynecol. 2019;54(4):517–23. doi: 10.1002/uog.20192 30549119

[19] Wang S, Wang Y, Feng L. Pregnancy outcomes following transvaginal cerclage for cervical insufficiency: Results from a single-center retrospective study. J Huazhong Univ Sci Technolog Med Sci. 2017;37(2):237–42. doi: 10.1007/s11596-017-1721-0 28397052

[20] Yao L-P, Yang Q, Pei J-D, Wu Y-L, Wan S, Chen Z-Q, et al. Ultrasound-Indicated Cervical Cerclage Efficacy Between 16 and 28 Weeks of Gestation in Twin Pregnancy: Retrospective Cohort Study. Int J Gen Med. 2022;15:2377. doi: 10.2147/IJGM.S341155 35264875 PMC8899099

[21] Henderson LK, Craig JC, Willis NS, Tovey D, Webster AC. How to write a Cochrane systematic review. Nephrol Ther. 2010;15(6):617–24. doi: 10.1111/j.1440-1797.2010.01380.x 20883282

[22] Dissemination C. Systematic reviews: CRD’s guidance for undertaking reviews in healthcare. York: University of York NHS Centre for Reviews & Dissemination. 2009.

[23] Stroup DF, Berlin JA, Morton SC, Olkin I, Williamson GD, Rennie D, et al. Meta-analysis of observational studies in epidemiology: a proposal for reporting. JAMA. 2000;283(15):2008–12. 10789670 10.1001/jama.283.15.2008

[24] Higgins J. Cochrane handbook for systematic reviews of interventions. Version 5.1. 0 (updated March 2011). The Cochrane Collaboration. www.cochrane-handbook.org. 2011.

[25] Page MJ, McKenzie JE, Bossuyt PM, Boutron I, Hoffmann TC, Mulrow CD, et al. The PRISMA 2020 statement: an updated guideline for reporting systematic reviews. BMJ. 2021 Mar 29;372:n71. doi: 10.1136/bmj.n71 ; PMCID: PMC8005924. 33782057 PMC8005924

[26] Higgins JP, Sterne JA, Savovic J, Page MJ, Hróbjartsson A, Boutron I, et al. A revised tool for assessing risk of bias in randomized trials. Cochrane Database Syst Rev. 2016;10(Suppl 1):29–31.

[27] Lo CK, Mertz D, Loeb M. Newcastle-Ottawa Scale: comparing reviewers’ to authors’ assessments. BMC Med Res Methodol. 2014 Apr 1;14:45. doi: 10.1186/1471-2288-14-45 ; PMCID: PMC4021422. 24690082 PMC4021422

[28] Puhan MA, Schünemann HJ, Murad MH, Li T, Brignardello-Petersen R, Singh JA, et al. A GRADE Working Group approach for rating the quality of treatment effect estimates from network meta-analysis. BMJ. 2014;349:g5630. doi: 10.1136/bmj.g5630 25252733

[29] Manzoli L, De Vito C, Salanti G, D’Addario M, Villari P, Ioannidis JP. Meta-analysis of the immunogenicity and tolerability of pandemic influenza A 2009 (H1N1) vaccines. PLoS ONE. 2011;6(9):e24384. doi: 10.1371/journal.pone.0024384 21915319 PMC3167852

[30] Hunter JP, Saratzis A, Sutton AJ, Boucher RH, Sayers RD, Bown MJ. In meta-analyses of proportion studies, funnel plots were found to be an inaccurate method of assessing publication bias. J Clin Epidemiol. 2014;67(8):897–903. doi: 10.1016/j.jclinepi.2014.03.003 24794697

[31] Flacco ME, Acuti Martellucci C, Baccolini V, De Vito C, Renzi E, Villari P, et al. COVID-19 vaccines reduce the risk of SARS-CoV-2 reinfection and hospitalization: Meta-analysis. Front Med. 2022;9:1023507. doi: 10.3389/fmed.2022.1023507 36438045 PMC9681813

[32] Deeks JJ, Higgins JPT, Altman DG. Cochrane Handbook for Systematic Reviews of Interventions Version 5.1.0. London: The Cochrane Collaboration; 2011.

[33] Qiu L, Lv M, Chen C, Li J, Zhao B, Luo Q. Efficacy of ultrasound-indicated cerclage in twin pregnancies: a retrospective case-control study matched by cervical length. Am J Obstet Gynecol MFM. 2023 Mar;5(3):100847. doi: 10.1016/j.ajogmf.2022.100847 36638868

[34] Liping Q, Min L, Chen C, Wang M, Luo Q. Efficacy of emergency cervical cerclage in twin pregnancies and factors affecting the clinical effects of emergency cerclage. J Matern Fetal Neonatal Med. 2023 Dec;36(1):2198632. doi: 10.1080/14767058.2023.2198632 37031971

[35] Zeng C, Liu X, Zhao Y, Pei C, Fu Y, Wang W, et al. Pregnancy outcomes and factors affecting the clinical effects of emergency cerclage in twin pregnancies with cervical dilation and prolapsed membranes. Int J Gynecol Obstet. 2022;157(2):313–21. doi: 10.1002/ijgo.13774 34076897

[36] Pan M, Zhang J, Zhan W, Ouyang X, Jiang X, Yang D. Physical examination-indicated cerclage in twin pregnancy: a retrospective cohort study. Arch Gynecol Obstet. 2021;303(3):665–76. doi: 10.1007/s00404-020-05777-y 32886234

[37] Han MN O’Donnell BE, Maykin MM, Gonzalez JM, Tabsh K, Gaw SL. The impact of cerclage in twin pregnancies on preterm birth rate before 32 weeks. J Matern Fetal Neonatal Med. 2019;32(13):2143–51. doi: 10.1080/14767058.2018.1427719 29363371 PMC6251764

[38] Qureshey EJ, Quiñones JN, Rochon M, Sarno A, Rust O. Comparison of management options for twin pregnancies with cervical shortening. J Matern Fetal Neonatal Med. 2022;35(1):39–45. doi: 10.1080/14767058.2019.1706477 31878811

[39] Abbasi N, Barrett J, Melamed N. Outcomes following rescue cerclage in twin pregnancies. J Matern Fetal Neonatal Med. 2018;31(16):2195–201. doi: 10.1080/14767058.2017.1338260 28597706

[40] Adams TM, Rafael TJ, Kunzier NB, Mishra S, Calixte R, Vintzileos AM. Does cervical cerclage decrease preterm birth in twin pregnancies with a short cervix? J Matern Fetal Neonatal Med. 2018;31(8):1092–8 doi: 10.1080/14767058.2017.1309021 28320233

[41] Houlihan C, Poon LC, Ciarlo M, Kim E, Guzman ER, Nicolaides KH. Cervical cerclage for preterm birth prevention in twin gestation with short cervix: a retrospective cohort study. Ultrasound Obstet Gynecol. 2016;48(6):752–6. doi: 10.1002/uog.15918 26990136

[42] Roman A, Rochelson B, Martinelli P, Saccone G, Harris K, Zork N, et al. Cerclage in twin pregnancy with dilated cervix between 16 to 24 weeks of gestation: retrospective cohort study. Am J Obstet Gynecol. 2016;215(1):98.e1–98.e11. doi: 10.1016/j.ajog.2016.01.172 26827881

[43] Roman A, Rochelson B, Fox NS, Hoffman M, Berghella V, Patel V, et al. Efficacy of ultrasound-indicated cerclage in twin pregnancies. Am J Obstet Gynecol. 2015;212(6):788.e1–6. 10.1016/j.ajog.2015.01.031 25637840

[44] Roman AS, Rebarber A, Pereira L, Sfakianaki AK, Mulholland J, Berghella V. The efficacy of sonographically indicated cerclage in multiple gestations. J Ultrasound Med. 2005;24(6):763–8. doi: 10.7863/jum.2005.24.6.763 15914680

[45] Newman RB, Krombach RS, Myers MC, McGee DL. Effect of cerclage on obstetrical outcome in twin gestations with a shortened cervical length. Am J Obstet Gynecol. 2002;186(4):634–40. doi: 10.1067/mob.2002.122126 11967484

[46] Althuisius SM, Dekker GA, Hummel P, Bekedam DJ, van Geijn HP. Final results of the Cervical Incompetence Prevention Randomized Cerclage Trial (CIPRACT): therapeutic cerclage with bed rest versus bed rest alone. Am J Obstet Gynecol. 2001;185(5):1106–12. doi: 10.1067/mob.2001.118655 11717642

[47] Rust OA, Atlas RO, Jones KJ, Benham BN, Balducci J. A randomized trial of cerclage versus no cerclage among patients with ultrasonographically detected second-trimester preterm dilatation of the internal os. Am J Obstet Gynecol. 2000 Oct;183(4):830–5. doi: 10.1067/mob.2000.109040 11035321

[48] Su J, Li D, Yang Y, Cao Y, Yin Z. Cerclage placement in twin pregnancies with cervical dilation: a systematic review and meta-analysis. J Matern Fetal Neonatal Med. 2022 Dec;35(25):9112–9118. doi: 10.1080/14767058.2021.2015577 34906023

[49] Li C, Shen J, Hua K. Cerclage for women with twin pregnancies: a systematic review and metaanalysis. Am J Obstet Gynecol. 2019 Jun;220(6):543–557.e1. 30527942 10.1016/j.ajog.2018.11.1105

[50] Li C, Hua K. Efficacy of physical examination-indicated cerclage in twin pregnancies compared with singleton pregnancies: a systematic review and meta-analysis. Minerva Obstet Gynecol. 2021 Feb;73(1):111–120. doi: 10.23736/S2724-606X.20.04518-9 32315128

[51] Saccone G, Rust O, Althuisius S, Roman A, Berghella V. Cerclage for short cervix in twin pregnancies: systematic review and meta-analysis of randomized trials using individual patient-level data. Acta Obstet Gynecol Scand. 2015 Apr;94(4):352–8. doi: 10.1111/aogs.12600 Epub 2015 Mar 1. . 25644964

[52] Berghella V, Ciardulli A, Rust OA, To M, Otsuki K, Althuisius S, et al. Cerclage for sonographic short cervix in singleton gestations without prior spontaneous preterm birth: systematic review and meta-analysis of randomized controlled trials using individual patient-level data. Ultrasound Obstet Gynecol. 2017 Nov;50(5):569–577. doi: 10.1002/uog.17457 Epub 2017 Oct 5. . 28295722

[53] Alfirevic Z, Stampalija T, Medley N. Cervical stitch (cerclage) for preventing preterm birth in singleton pregnancy. Cochrane Database Syst Rev. 2017 Jun 6;6(6):CD008991. doi: 10.1002/14651858.CD008991.pub3 ; PMCID: PMC6481522. 28586127 PMC6481522

[54] Khalil A, Rodgers M, Baschat A, Bhide A, Gratacos E, Hecher K, et al. ISUOG Practice Guidelines: role of ultrasound in twin pregnancy. Ultrasound Obstet Gynecol. 2016 Feb;47(2):247–63. doi: 10.1002/uog.15821 Erratum in: Ultrasound Obstet Gynecol. 2018 Jul;52(1):140. . 26577371

[55] Conde-Agudelo A, Romero R. Cervicovaginal fetal fibronectin for the prediction of spontaneous preterm birth in multiple pregnancies: a systematic review and meta-analysis. J Matern Fetal Neonatal Med. 2010;23(12):1365–76. doi: 10.3109/14767058.2010.499484 21067303 PMC3418880

[56] Conde-Agudelo A, Romero R, Hassan SS, Yeo L. Transvaginal sonographic cervical length for the prediction of spontaneous preterm birth in twin pregnancies: a systematic review and metaanalysis. Am J Obstet Gynecol. 2010;203(2):128.e1–12. doi: 10.1016/j.ajog.2010.02.064 20576253 PMC3147231

[57] Kindinger LM, Poon LC, Cacciatore S, MacIntyre DA, Fox NS, Schuit E, et al. The effect of gestational age and cervical length measurements in the prediction of spontaneous preterm birth in twin pregnancies: An individual patient level meta-analysis. BJOG. 2016;123:877–884. doi: 10.1111/1471-0528.13575 26333191

