# Supplementary material for: Cervical cerclage for prevention of preterm birth and adverse perinatal outcome in twin pregnancies with short cervical length or cervical dilatation: A systematic review and meta-analysis
Source: PLoS Med. 2023 Aug 3;20(8):e1004266. doi: 10.1371/journal.pmed.1004266 (PMC10456178; doi:10.1371/journal.pmed.1004266)
Supplement: S1 Table — (DOCX) [file pmed.1004266.s001.docx]

**Supplementary Table 1.** Search strategy.

**Search Strategy**

**Databases:** Embase, MEDLINE

(results downloaded on 6 July 2022)

| **Set#** | **Searched for** | **Results** |
| --- | --- | --- |
| S1 | MESH.EXACT("Cerclage, Cervical") OR EMB.EXACT("cerclage") OR EMB.EXACT("uterine cervix cerclage") or ti,ab,if(cerclage[*1] or "cervical stitch*" or "cervix stitch*") | 10442 |
| S2 | MESH.EXACT("Pregnancy, Twin") OR MESH.EXACT("Pregnancy, Multiple") OR MESH.EXACT("Twin Studies as Topic") OR MESH.EXACT.EXPLODE("Twins") OR rtype.exact("Twin Study") OR MESH.EXACT("Diseases in Twins") OR MESH.EXACT.EXPLODE("Twinning, Embryonic") | 46145 |
| S3 | EMB.EXACT("twin pregnancy") OR EMB.EXACT("multiple pregnancy") OR EMB.EXACT.EXPLODE("twins") OR EMB.EXACT("twin study") OR EMB.EXACT.EXPLODE("twin zygosity") OR EMB.EXACT.EXPLODE("twinning") | 81003 |
| S4 | ti,ab,if(twin or twins or twinning) or ti,ab,if(multiple near/5 (pregnan[*4] or gestation[*1] or birth[*1])) | 199143 |
| S5 | s1 and (s2 or s3 or s4) | 1012 |
| S6 | rtype.exact("Clinical Trial, Phase III" OR "Randomized Controlled Trial" OR "Controlled Clinical Trial" OR "Clinical Trial, Phase IV" OR "Clinical Trial, Phase I" OR "Meta-Analysis" OR "Clinical Trial" OR "Clinical Trial, Phase II" OR "Pragmatic Clinical Trial" OR "Equivalence Trial" OR "Adaptive Clinical Trial" OR "Multicenter Study" OR "Systematic Review") | 1408322 |
| S7 | MESH.EXACT.EXPLODE("Clinical Trials as Topic") OR MESH.EXACT.EXPLODE("Meta-Analysis as Topic") OR MESH.EXACT("Random Allocation") OR MESH.EXACT("Single-Blind Method") OR MESH.EXACT("Cross-Over Studies") OR MESH.EXACT("Double-Blind Method") OR MESH.EXACT("Placebos") OR MESH.EXACT("Multicenter Studies as Topic") OR MESH.EXACT("Systematic Reviews as Topic") OR MESH.EXACT("Review Literature as Topic") | 714917 |
| S8 | EMB.EXACT("phase 1 clinical trial") OR EMB.EXACT("phase 3 clinical trial") OR EMB.EXACT.EXPLODE("randomized controlled trial") OR EMB.EXACT("phase 2 clinical trial") OR EMB.EXACT("phase 4 clinical trial") OR EMB.EXACT("controlled clinical trial") OR EMB.EXACT.EXPLODE("clinical trial") OR EMB.EXACT("triple blind procedure") OR EMB.EXACT("double blind procedure") OR EMB.EXACT("crossover procedure") OR EMB.EXACT("single blind procedure") OR EMB.EXACT.EXPLODE("randomization") OR EMB.EXACT("placebo") OR EMB.EXACT("multicenter study") OR EMB.EXACT.EXPLODE("meta analysis") OR EMB.EXACT("systematic review") OR EMB.EXACT.EXPLODE("clinical trial (topic)") OR EMB.EXACT("meta analysis (topic)") OR EMB.EXACT("systematic review (topic)") OR EMB(ct) | 2924309 |
| S9 | ti,ab,if("meta analy[*3]" or metaanaly[*3] or "systematic review[*1]" or "systematic overview[*1]" or "integrative review[*1]" or "integrative research review[*1]" or "rapid review[*1]" or "umbrella review[*1]" or trial[*1] or ((singl[*4] or doubl[*4] or trebl[*4] or tripl[*4]) near/5 (blind[*4] or mask[*4])) or rct[*1] or randomized or randomised or randomization or randomisation or randomly or crossover or "cross over" or placebo[*1] or (random near/3 allocat[*4])) | 5831460 |
| S10 | pub.exact("Cochrane Database of Systematic Reviews" OR "Cochrane database of systematic reviews (Online)" OR "The Cochrane database of systematic reviews") | 39805 |
| S11 | MESH.EXACT("Cohort Studies") or MESH.EXACT.EXPLODE("Longitudinal Studies") or MESH.EXACT("Follow-Up Studies") or MESH.EXACT("Retrospective Studies") or MESH.EXACT("Case-Control Studies") or MESH.EXACT("Cross-Sectional Studies") or MESH.EXACT("Prospective Studies") or MESH.EXACT("Controlled Before-After Studies") OR MESH.EXACT("Historically Controlled Study") or MESH.EXACT("Interrupted Time Series Analysis") or MESH.EXACT("Epidemiologic Studies") or MESH.EXACT("Pilot Projects") or MESH.EXACT("Feasibility Studies") OR MESH.EXACT("Observational Studies as Topic") or MESH.EXACT("Clinical Studies as Topic") | 3112798 |
| S12 | rtype.exact("Observational Study" or "Comparative Study" or "Clinical Study") | 2033836 |
| S13 | EMB.EXACT.EXPLODE("longitudinal study") or EMB.EXACT.EXPLODE("case control study") or EMB.EXACT("prospective study") or EMB.EXACT("major clinical study") or EMB.EXACT("retrospective study") or EMB.EXACT("clinical study") or EMB.EXACT("cross-sectional study") or EMB.EXACT("cohort analysis") or EMB.EXACT("observational study") or EMB.EXACT("community trial") or EMB.EXACT("open study") or EMB.EXACT("family study") or EMB.EXACT("pilot study") or EMB.EXACT("comparative study") or EMB.EXACT("controlled study") or EMB.EXACT("follow up") or EMB.EXACT("evaluation and follow up") | 14118286 |
| S14 | ti,ab,if(prospective[*2] or retrospective[*2] or "follow up" or followup or longitudinal[*2] or "case control[*4]" or "cross sectional[*2]" or observation[*2] or (case[*1] near/5 series)) or ti,ab,if((cohort or epidemiologic[*4]) near/5 (study or studies or analy[*3])) | 11019700 |
| S15 | s5 and (s6 or s7 or s8 or s9 or s10 or s11 or s12 or s13 or s14) | 684 |
| S16 | s5 not (s15 or rtype.exact("Case Reports") or EMB.EXACT("case report") or EMB.EXACT.EXPLODE("case study") or ti,ab,if("case study" or "case report")) | 227 |
| S17 | s15 or s16 | 864 |
| S18 | (s15 or s16) and human(yes) | 822 |
| S19 | (s15 or s16) not (human(yes) or animal(yes) or EMB.EXACT("nonhuman")) | 66 |
| **S20** | **s18 or s19** | **982** |

**Databases:** Cochrane Library

(results downloaded on 6 July 2022)

| **ID** | **Search** | **Hits** |
| --- | --- | --- |
| #1 | MeSH descriptor: [Cerclage, Cervical] this term only | 62 |
| #2 | (cerclage* or (cervical next stitch*) or (cervix next stitch*)):ti,ab,kw (Word variations have been searched) | 452 |
| #3 | MeSH descriptor: [Pregnancy, Twin] this term only | 76 |
| #4 | MeSH descriptor: [Pregnancy, Multiple] this term only | 198 |
| #5 | MeSH descriptor: [Twin Studies as Topic] this term only | 5 |
| #6 | MeSH descriptor: [Twins] explode all trees | 196 |
| #7 | MeSH descriptor: [Diseases in Twins] this term only | 42 |
| #8 | MeSH descriptor: [Twinning, Embryonic] explode all trees | 0 |
| #9 | (twin or twins or twinning):ti,ab,kw (Word variations have been searched) | 2062 |
| #10 | (multiple near/5 (pregnan* or gestation* or birth*)):ti,ab,kw (Word variations have been searched) | 2023 |
| **#11** | **(#1 or #2) and (#3 or #4 or #5 or #6 or #7 or #8 or #9 or #10)** | **81** |

- The Cochrane Database of Systematic Reviews (CDSR, Cochrane Reviews: Issue 7 of 12, July 2022) (5 references)
- The Cochrane Central Register of Controlled Trials (CENTRAL, Trials: Issue 7 of 12, July 2022) (71 references)
