# Supplementary material for: Cervical cerclage for prevention of preterm birth and adverse perinatal outcome in twin pregnancies with short cervical length or cervical dilatation: A systematic review and meta-analysis
Source: PLoS Med. 2023 Aug 3;20(8):e1004266. doi: 10.1371/journal.pmed.1004266 (PMC10456178; doi:10.1371/journal.pmed.1004266)
Supplement: S3 Table — (DOCX) [file pmed.1004266.s003.docx]

**Supplementary Table 3.** Excluded studies and reason for the exclusion.

| **Author** | **Year** | **Title** | **Reason for the exclusion** |
| --- | --- | --- | --- |
| Zhao | 2023 | Impact of prolonged use of adjuvant tocolytics after cervical cerclage on late abortion and premature delivery | Only women undergoing cerclage were included in this series |
| Sanchez-Ramos | 2022 | Cerclage placement in twin pregnancies with short or dilated cervix does not prevent preterm birth: a fragility index assessment | Research letter, no original data included |
| Cai | 2022 | Correlation Between Clinical Factors and Pregnancy Outcome Following Repeat Cerclage: A Retrospective Analysis of a Chinese Population | Only women with repeated cerclage were included in this study |
| Groussolles | 2022 | Arabin pessary to prevent adverse perinatal outcomes in twin pregnancies with a short cervix: a multicenter randomized controlled trial (PESSARONE) | RCT on pessary |
| Park | 2022 | Successful delayed delivery of the second twin by evacuating the cord prolapsed first fetus and emergent cerclage: a report of 2 cases | Case report on cerclage after the delivery of one twin |
| Putora | 2022 | Progesterone, cervical cerclage or cervical pessary to prevent preterm birth: a decision-making analysis of international guidelines | No original data included |
| Ekici | 2022 | Cervical cerclage in twin pregnancies: obstetric and neonatal outcomes | Only women undergoing cerclage were included in this series |
| Park | 2022 | Ultrasound-Indicated Cerclage in Twin Pregnancies: A Cohort Study | Study comparing the efficacy of cervical cerclage in twin vs singleton gestations; no control group of twins managed expectantly was included in this series |
| Hsu | 2022 | Rescue Cervical Cerclage for Protruding Amniotic Sac: A Retrospective Analysis of Clinical Efficacy | Only 4 twin pairs included in this series |
| Cao | 2022 | The comparative analysis of laparoscopic or transvaginal cerclage in pregnancies with cervical insufficiency: a retrospective cohort study | No data for the outcomes explored in this systematic review |
| Kumar | 2022 | A retrospective study analyzing indications and outcomes of mid-trimester emergency cervical cerclage in a tertiary care perinatal centre over half a decade | No data for the outcomes explored in this systematic review |
| Ponce | 2022 | Latency to delivery in physical examination‐indicated cerclage in twins is similar to that in singleton pregnancies | Study comparing cervical cerclage in twins vs singletons |
| Perales-Marin | 2021 | Cerclage in twin gestations: the need to consider the effect of antibiotics and indomethacin | Letter to Editor, no original data included |
| Freegard | 2021 | Emergency cervical cerclage in twin and singleton pregnancies with 0‐mm cervical length or prolapsed membranes | No data for the outcomes explored in this systematic review |
| Lee | 2021 | Severe maternal morbidity associated with cerclage use in pregnancy. | All twin pregnancies were included in this study and the authors did not report the outcome in women with short cervix or cervical dilatation |
| Wei | 2021 | A comparison of pregnancy outcome of emergency modified transvaginal cervicoisthmic cerclage performed in twin and singleton pregnancies | Study comparing cervical cerclage in twins vs singletons |
| Hajizadeh | 2020 | Comparison of cerclage and pessary in prevention of preterm birth in twin pregnancies | The control group includes women undergoing another intervention (pessary) |
| Zhou | 2020 | Effects of vaginal microbiota and cervical cerclage on obstetric outcomes of twin pregnancies with cervical incompetence: a retrospective study | The authors stratified their analysis according to the presence or not of cervical incompetence. However, they defined cervical incompetence as cervical shortening, dilatation, previous history of pregnancy loss or preterm birth in the second trimester were suggestive of CIC. Our study population does not consider a prior history of PTB or pregnancy loss as inclusion criteria for the analysis |
| Frenken | 2020 | Cervical cerclage for prevention of preterm birth: the results from A 20-year cohort | No data for the outcomes explored in this systematic review |
| Hajizadeh | 2020 | Comparison of cerclage and pessary in prevention of preterm birth in twin pregnancies | All twin pregnancies were included in this study and the authors did not report the outcome in women with short cervix or cervical dilatation |
| Alkhaja | 2020 | The Effectiveness of Cervical Cerclage in Preventing Preterm Labor in a Twin Pregnancy | No data for the outcomes explored in this systematic review |
| Debieve | 2019 | Transabdominal cerclage for cervical insufficiency in twins: series of seven cases and literature review | Only cases undergoing transabdominal cerclage were included in this series |
| Barbosa | 2019 | Twin pregnancies treated with emergency or ultrasound-indicated cerclage to prevent preterm births | No control group included |
| Fichera | 2019 | The use of ultrasound‐indicated cerclage or cervical pessary in asymptomatic twin pregnancies with a short cervix at midgestation | No control group included |
| Cilingir | 2018 | Emergency cerclage in twins during mid gestation may have favorable outcomes: Results of a retrospective cohort | No control group included |
| Chun | 2018 | Effects of emergency cerclage on the neonatal outcomes of preterm twin pregnancies compared to preterm singleton pregnancies: A neonatal focus | Study comparing cervical cerclage in twins vs singletons |
| Park | 2018 | Outcomes of physical examination-indicated cerclage in twin pregnancies with acute cervical insufficiency compared to singleton pregnancies | Study comparing cervical cerclage in twins vs singletons |
| Matsui | 2017 | Preliminary preventive protocol from ﬁrst trimester of pregnancy to reduce preterm birth rate for dichorionicediamniotic twins | It was not possible to extrapolate data for twins with short cervix or cervical dilatation |
| Chavan | 2016 | Comparison of perinatal outcome in twin pregnancy with and without cervical cerclage | It was not possible to extrapolate data for twins with short cervix or cervical dilatation |
| Chavan | 2016 | Comparison of perinatal outcome in twin pregnancy with and without cervical cerclage | No data for the outcomes explored in this systematic review |
| bernabeu | 2016 | Physical examination-indicated cerclage in singleton and twin pregnancies: maternal–fetal outcomes | Study comparing cervical cerclage in twins vs singletons |
| Holcomb | 2014 | The effect of cerclage in twin gestations with short cervix: a Bayesian evaluation | No real cases included in this study |
| Galyean | 2014 | Removal versus retention of cerclage in preterm premature rupture of membranes: A randomized controlled trial | No data for the outcomes explored in this systematic review |
| Rebarber | 2014 | Outcomes of emergency or physical examination-indicated cerclage in twin pregnancies compared to singleton pregnancies | Study comparing cervical cerclage in twins vs singletons |
| Zanardini | 2013 | Cervical cerclage in twin pregnancies | No control group included |
| Aguilera | 2013 | Emergency Cerclage Placement in Multifetal Pregnancies with a Dilated Cervix and Exposed Membranes: Case Series | No control group included |
| Roman | 2013 | Prophylactic cerclage in the management of twin pregnancies | No data for the outcomes explored in this systematic review |
| Levin | 2012 | Outcomes of Mid-Trimester Emergency Cerclage in Twin Pregnancies | No control group included |
| Gupta | 2010 | Emergency cervical cerclage: predictors of success | No data for the outcomes explored in this systematic review |
| Eskandar | 2007 | Cervical cerclage for prevention of preterm birth in women with twin pregnancy | No data for the outcomes explored in this systematic review |
| Berghella | 2004 | Cerclage for prevention of preterm birth in women with a short cervix found on transvaginal ultrasound examination: a randomized trial | Case series. Small number of included cases (below the threshold for inclusion in the present review) |
| Parilla | 2003 | The prevalence and timing of cervical cerclage placement in multiple gestations | No data for the outcomes explored in this systematic review |
