# Supplementary material for: Cervical cerclage for prevention of preterm birth and adverse perinatal outcome in twin pregnancies with short cervical length or cervical dilatation: A systematic review and meta-analysis
Source: PLoS Med. 2023 Aug 3;20(8):e1004266. doi: 10.1371/journal.pmed.1004266 (PMC10456178; doi:10.1371/journal.pmed.1004266)
Supplement: S4 Table — (DOCX) [file pmed.1004266.s004.docx]

**Supplementary Table 4**. Women with a reduced cervical length on ultrasound and/or cervical dilatation at examination: Results of the proportion meta-analyses estimating the pooled rates of each clinical outcome in women undergoing cerclage versus women not undergoing cerclage.

|  | **Cerclage** | |  | **No cerclage** | |
| --- | --- | --- | --- | --- | --- |
| *Outcomes* | **n / N** | **Pooled %**  **(95% CI)** |  | **n / N** | **Pooled %**  **(95% CI)** |
|  |  |  |  |  |  |
| PTB<34 week | 327/690 | 48.0 (38.3, 57.7) |  | 353/530 | 71.3 (57.0, 83.9) |
| *Reduced length only - by cervical length:* |  |  |  |  |  |
| - <15mm | 101/199 | 44.0 (28.5, 60) |  | 121/154 | 79.4 (72.2, 75.8) |
|  |  |  |  |  |  |
| 15-25mm | 61/159 | 35.5 (21.8, 50.4) |  | 63/140 | 53.1 (29.4, 76.1) |
|  |  |  |  |  |  |
| pPROM | 162/606 | 28.0 (22.2, 34.1) |  | 469/498 | 37.0 (23.2, 52.0) |
|  |  |  |  |  |  |
| Chorioamnionitis | 47/461 | 15.3 (5.3, 28.4) |  | 29/306 | 11.9 (1.5, 28.0) |
|  |  |  |  |  |  |
| Cesarean delivery | 346/509 | 67.3 (60.1, 74.1) |  | 215/397 | 46.0 (29.7, 62.8) |
|  |  |  |  |  |  |
| Perinatal loss | 202/1538 | 11.1 (6.5, 16.7) |  | 347/1204 | 29.2 (15.7, 44.9) |
|  |  |  |  |  |  |
| Composite adverse neonatal outcome | 559/1196 | 43.7 (34.4, 54.4) |  | 460/745 | 67.6 (53.4, 80.3) |
|  |  |  |  |  |  |
| 5-minute Apgar score<7 | 125/563 | 21.6 (13.9, 30.5) |  | 156/411 | 45.4 (24.6, 67.0) |
|  |  |  |  |  |  |
| Respiratory distresss syndrome | 85/327 | 26.9 (15.8, 39.6) |  | 73/193 | 43.3 (11.3, 78.4) |
|  |  |  |  |  |  |
| Sepsis | 17/241 | 6.5 (3.1, 10.9) |  | 36/117 | 19.7 (10.9, 30.0) |
|  |  |  |  |  |  |
| Intraventricular hemorrhage grade III-IV | 20/327 | 5.5 (1.5, 11.4) |  | 44/193 | 19.1 (1.2, 47.4) |
|  |  |  |  |  |  |
| Necrotizing enterocolitis | 13/327 | 3.0 (0.44, 7.1) |  | 24/193 | 9.6 (0.31, 25.9) |
|  |  |  |  |  |  |
| Retinopathy of prematurity | 14/227 | 5.8 (1.8, 11.5) |  | 17/160 | 10.9 (0.61, 28.3) |
|  |  |  |  |  |  |
| Birthweight <1500 grams | 282/863 | 33.2 (25.6, 41.2) |  | 357/691 | 65.0 (45.2, 82.3) |
|  |  |  |  |  |  |
| Admission to NICU | 613/114 | 55.8 (45.3, 66.0) |  | 502/721 | 80.7 (69.0, 90.2) |
|  |  |  |  |  |  |

n/N: Number of women with the outcome / Total number of women. CI: confidence interval; pPROM: Preterm prelabour rupture of the membranes; NICU: neonatal Intensive Care Unit.
