# Supplementary material for: Cervical cerclage for prevention of preterm birth and adverse perinatal outcome in twin pregnancies with short cervical length or cervical dilatation: A systematic review and meta-analysis
Source: PLoS Med. 2023 Aug 3;20(8):e1004266. doi: 10.1371/journal.pmed.1004266 (PMC10456178; doi:10.1371/journal.pmed.1004266)
Supplement: S1 Fig — (DOCX) [file pmed.1004266.s006.docx]

**S1 Fig**. Funnel plot of the effect estimates vs their standard errors (outcome: Risk of preterm birth <34th week in women undergoing cerclage versus no cerclage - Women with a reduced cervical length on ultrasound and/or cervical dilatation at examination).

**Egger: bias = 0.23 (95% CI = -2.12, 2.57) p = 0.8**
