# Supplementary material for: Cervical cerclage for prevention of preterm birth and adverse perinatal outcome in twin pregnancies with short cervical length or cervical dilatation: A systematic review and meta-analysis
Source: PLoS Med. 2023 Aug 3;20(8):e1004266. doi: 10.1371/journal.pmed.1004266 (PMC10456178; doi:10.1371/journal.pmed.1004266)
Supplement: S4 Fig — (DOCX) [file pmed.1004266.s009.docx]

**S4 Fig**. Funnel plot of the effect estimates vs their standard errors (outcome: Gestational age in women undergoing cerclage versus no cerclage - Women with a reduced cervical length on ultrasound and/or cervical dilatation at examination).

**Egger: bias = 1.01 (95% CI = -2.51, 4.53) p = 0.6**
