# Supplementary material for: Cervical cerclage for prevention of preterm birth and adverse perinatal outcome in twin pregnancies with short cervical length or cervical dilatation: A systematic review and meta-analysis
Source: PLoS Med. 2023 Aug 3;20(8):e1004266. doi: 10.1371/journal.pmed.1004266 (PMC10456178; doi:10.1371/journal.pmed.1004266)
Supplement: Attachment — Submitted filename: dantonio.pdf [file pmed.1004266.s013.pdf]

This paper describes a systematic review with meta–analysis of studies of cervical cerclage in twin pregnancies and its relationship with various adverse outcomes.

I was asked for a statistical report and I interpret that to include all aspects of the design and conduct of the study.

# Points of detail

**Page 2** Is it true that the pregnancies 'were allocated to cerclage [. . . ]'? Few of the studies found here were trials where allocation is performed. The same wording is used elsewhere.

**Page 5** The section on language is not clear to me. Do the authors mean that they excluded all studies in a language other than English? The message I draw from Grégoire et al. (1995); Moher et al. (1996); Egger et al. (1997); Jüni et al. (2002); Konno et al. (2020); Nussbaumer-Streit et al. (2020); Bachelet et al. (2021) is that bias is possible although it is hard to predict in advance the extent or direction of it. I would not necessarily commit the authors to translate everything but at least we should know what we are missing. (Jackson et al., 2019) has produced some evidence about the successful use of Google Translate in systematic reviews.

**Page 7** If authors were asked to supply extra data then I think that (a) we should be told which items we are not going to be able to find in the original publications (b) the people who supplied it should be acknowledged.

**Page 8** Confidence intervals for $I^2$ would also be helpful to reveal our uncertainty about the heterogeneity. I know we did not use to use them but I am persuaded by Ioannidis et al. (2007) that we should.

**Page 8** Since the various measures of risk are rather different I would question the wisdom of comparing them. At least include a moderator for measure to account for differences.

**Page 8** What funnel plots and regression tests hope to identify is small study effects. Calling it publication bias implies you know the aetiology.

**Page 9** The authors have helpfully provided a copy of their data but it seems to only contain four randomised trials not five: Roman 2020, Berghellla 2004, Rust 2000, Althuisius 2001.

**Page 10** I would suggest that the primary outcome should be reported first not hidden in the middle of the secondaries.

**Page 11 onwards** Some of these have lost their effect size and its confidence interval. Finding that the confidence interval includes the null does not show no difference, it shows that the study was unable to show a difference.

**Page 12** I am not sure what the relevance of comparing singleton pregnancies with twin pregnancies is. This needs clarifying.

**Page 14** The authors state that 'most of the included studies were not sufficiently powered'. Surely that is why we do meta–analysis, to draw conclusions from the totality of evidence?

**Figure 1** It would be better to clarify in this how many articles are relevant for the analysis of twin pregnancies and how many for the analysis of single versus twin.

# Points of more substance

## Mixing trials and observational

I think most people would view trials as providing better information than observational studies not least in the avoidance of selection bias. The authors have not put forward a convincing justification for presenting them mixed together. I took the data which the authors have kindly provided in machine–readable format and ran a meta–regression. I calculated the odds ratios from the figures for less than 34 weeks, the primary outcome. I included a moderator for study type and fitted the model in R using the metafor package and estimating $\tau^2$ with REML.. The relevant information was an effect of 0.35 (95% ci 0.20 to 0.59) with a relative odds ratio for trials compared to observational of 5.50 (1.07 to 28.20). Since that does not include the null value (unity) I conclude that there is a real difference between the two study types with the observational studies being more optimistic. My conclusion would be that the observational studies are biased, probably because of selection bias perhaps caused by bias by indication.

Obviously the authors will wish to confirm my analysis with their own copy of the data and their software. Different software often gives different results.

## Small study effects

The authors refer to the presence of small study effects, for instance on page 13, although without presenting any evidence of them. Figure 1 shows the funnel plot from the meta–regression using study type as a moderator.

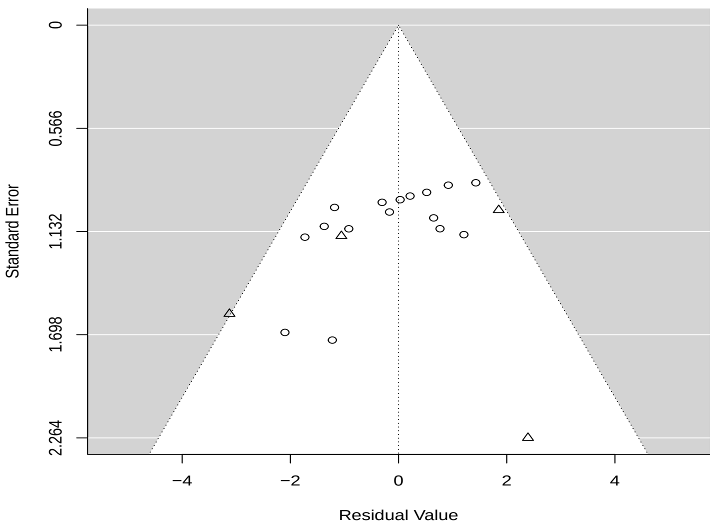


Figure 1: Funnel plot from the meta–regression

Note that with a model using a moderator the appropriate variable for the $x$–axis is the residuals from the model. In the plot I have marked the trials as triangles and the observational studies as circles. I do not see any noticeable evidence here of asymmetry bearing in mind the findings of Terrin et al. (2005) that researchers are poor at identifying asymmetry from funnel plots.

# Summary

As is clear I do not draw the same conclusions as the authors from their review. At the moment the trial evidence seems to be based on 78 pregnancies for the primary outcome. Given the conflict between those trials and the observational studies it would seem that what is needed is more trials. I have never been involved in an obstetric trial but I imagine the small number of participants reflects genuine difficulty in recruiting women into such trials but at the moment there must be genuine doubt about the advice they are to be offered.

Michael Dewey

# References

V C Bachelet, M S Navarrete, C Barrera-Riquelme, V A Carrasco, M Dallaserra, R A Díaz, A A Ibarra, F J Lizana, N Mezza-Ducaud, M G Saavedra, C Tapia-Davegnoa, A F Vergara, and J Villanueva. A multiyear systematic survey of the quality of reporting for randomised trials in dentistry, neurology, and geriatrics published in journals of Spain and Latin America. *BMC Medical Research Methodology*, 21(153), 2021.

M Egger, T Zellweger-Zähner, M Schneider, C Junker, C Lengeler, and G Antes. Language bias in randomised controlled trials published in English and German. *Lancet*, 350:326–329, 1997.

G Grégoire, F Derderian, and J Le Lorier. Selecting the language of the publications included in a meta-analysis: is there a tower of Babel bias? *Journal of Clinical Epidemiology*, 48(1):159–163, 1995.

J Ioannidis, N Patsopoulos, and E Evangelou. Uncertainty in heterogeneity estimates in meta–analyses. *British Medical Journal*, 335:914–916, 2007.

J L Jackson, A Kuriyama, A Anton, A Choi, J-P Fournier, A-K Geier, F Jacquerioz, D Kogan, and R Sun. The accuracy of Google Translate for abstracting data from non–English–language trials for systematic reviews. *Annals of Internal Medicine*, 171:677–679, 2019.

P Jüni, F Holenstein, J Sterne, C Bartlett, and M Egger. Direction and impact of language bias in meta–analyses of controlled trials: empirical study. *International Journal of Epidemiology*, 31:115–123, 2002.

K Konno, M Akasaka, C Koshida, N Katayama, N Osada, R Spake, and T Amano. Ignoring non–English–language studies may bias ecological meta–analyses. *Ecology and Evolution*, 10:6373–6384, 2020.

D Moher, P Fortin, A R Jadad, P Jüni, T Klassen, J Le Lorier, A Liberati, K Linde, and A Penna. Completeness of reporting of trials published in languages other than English: implications for conduct and reporting of systematic reviews. *Lancet*, 347:363–366, 1996.

B Nussbaumer-Streit, I Klerings, A I Dobrescu, E Persad, A Stevens, C Garrity, C Kamel, L Affengruber, V J King, and G Gartlehner. Excluding non–english publications from evidence–syntheses did not change conclusions: a meta–epidemiological study. *Journal of Clinical Epidemiology*, 118:42–54, 2020.

N Terrin, C H Schmid, and J Lau. In an empirical evaluation of the funnel

plot, researchers could not visually identify publication bias. *Journal of Clinical Epidemiology*, 58:894–901, 2005.
