# Supplementary material for: Cervical cerclage for prevention of preterm birth and adverse perinatal outcome in twin pregnancies with short cervical length or cervical dilatation: A systematic review and meta-analysis
Source: PLoS Med. 2023 Aug 3;20(8):e1004266. doi: 10.1371/journal.pmed.1004266 (PMC10456178; doi:10.1371/journal.pmed.1004266)
Supplement: Attachment — Submitted filename: Reply_to_rviewers-and_editors_queries.docx [file pmed.1004266.s014.docx]

Dear Editors,

We would like to thank you for the opportunity to provide a revised version of this study. Please note that:

1. We have now re-run all the statistical analyses reporting the results for RCT and observational studies separately.
2. Updated the search with no language restrictions.
3. Changed the inclusion criteria and focused on well specific population of twin pregnancies, those with short cervical length at ultrasound or cervical dilatation at physical examination, as requested by the editor
4. Extensively revised the manuscript

Requests from the editors:

GENERAL
Please respond to all editor and reviewer comments detailed below, in full.
Thank you for reporting your SRMA according to PRISMA. Please provide the completed PRISMA checklist.
When completing the checklist, please use section and paragraph numbers, rather than page or line numbers as these often change in the event of publication.
Please modify the statement in the Methods section to signpost the checklist: "This study is reported as per the Preferred Reporting Items for Systematic Reviews and Meta-Analyses (PRISMA) guideline (S1 Checklist)." Or similar.

***Reply:*** *Thank you for your suggestion. This has been done now.*

*** The reviewers and academic editor have raised specific concerns regarding the design and methodological approaches used in your study. Please see below for specific comments which we agree with. ***

COMMENTS FROM THE ACADEMIC EDITOR
1. The paper cannot be published in its present form.

***Reply:*** *thank you for your comment. We have now replied to all the editor’s and reviewers’ queries. We hope that you will find the manuscript improved.*

2. The authors seem to interpret their results as making the case for cerclage in all twins with a short cervix (<15mm). But the vast bulk of the data come from observational studies and, as the stats review points out, the RCTs differ from the observational studies and there is obviously huge potential for bias when comparing women having and not having an intervention in an observational study design.

***Reply:*** *Thank you for your comment. We have now completely re-run the analysis, after having updated the search, providing separate analyses for RCT and observational studies too. We have also tempered our conclusions derived from GRADE assessment.*

3. Even the RCT data are poor. Much of it is old, and even the more recent Roman paper only included 34 women and the intervention also included indomethacin and antibiotics, so it is not clear that any effect in this group was due to cerclage.

*Reply: Thank you very much for your comment. We have now stressed this limitation in the discussion even further and also performed sub-group analyses including RCT and observational studies separately. The text now states: “The most significant limitation of the present systematic review relies on the inclusion of mainly observational studies. The large majority of RCT were old, with a very small number of cases and heterogeneous population of twin pregnancies, thus considerably limiting the robustness of their findings. Only one RCT was published in the recent past, showing a potential beneficial role of cerclage in women with cervical dilatation. However, even this trial, despite being power for its primary outcome, was limited by a very small number of included cases and also by potential confounders such as the use of indomethacin and antibiotics in the intervention arm”.*

I think that this paper best serves to provide the data required to design the RCT that could change clinical practice. I recommend:
1. That they need to remove all analyses where they just lump all twin pregnancies together, i.e. there should be separate analysis of those where cerclage was indicated and those where it was not and no analysis where these two groups are pooled.

***Reply:*** *Thank you for your comment. As you suggested, we have now removed the analysis on the role of cerclage in an unselected population of twins and in twins vs singletons. We have also changed the title so that we can specifically focus on twin with require a clinical indication for cervical cerclage, such as those with shorth cervical length or cervical dilatation. The title now states:* *Cervical cerclage for prevention of preterm birth and adverse perinatal outcome in twin pregnancies with short cervical length or cervical dilatation: a systematic Review and Meta-analysis”*

2. That they provide separate analysis of RCTs and observational studies and they should formally test for differences in the estimate of effect size between the two groups.

***Reply:*** *Thank you for your comment. We have now provided two separate analyses including RCT and observational studies separately.*

3. They re-focus the discussion around how their analyses inform a trial - inclusion criteria, comparison group, primary outcome, sample size etc.

***Reply:*** *Thank you for your comment. We have now modified the discussion re-focusing the study findings on the need for a RCT. The text now states:”* *Although the findings from this meta-analysis suggest a potential beneficial role of cervical cerclage in reducing the risk of PTB and improving neonatal outcome in women at risk, the inclusion of mainly observational studies significantly affect the robustness of the results and should be confirmed in adequately powered RCTs. Only three RCTs were included, with a very small number of women allocated to cerclage or standard care. Ideally, a RCT on the role of cerclage in twin pregnancies should include women with sohrt cervix on ultarsound or cervical dialattion separately and be powered to maternal and neonatal outcomes. Furthemore, this trial should share an objective protocol for prenatal assessment of women at risk and management of women before and after cerclage placement, including timing of ultarsoudn assessment pof cervical length by ultrasound and preventive startegies for PTB, including progesterone and tocolysis”.*

ABSTRACT
Please ensure that your abstract is reported according to PRISMA for abstracts, following the PLOS Medicine abstract structure (Background, Methods and Findings, Conclusions) <http://www.plosmedicine.org/article/info:doi/10.1371/journal.pmed.1001419>
Abstract methods and findings: this reads very nicely but does seem to be rather long. Perhaps fewer of the secondary outcomes could be listed (those considered most important) and the same examples then evidenced with statistical information.
Please provide the beginning and dates of the (updated) search, data sources, number of studies included, types of study designs included, and synthesis/appraisal methods
***Reply:*** *Thank you for your suggestion. We have now completely re-written the abstract following the Prisma statement.*

Line 85: “The level of evidence was downgraded…” the sentence is rather vague (“imprecision of results” and “indirectness of evidence”) and somewhat discredits your own data. Suggest revising this statement and concluding this section with a sentence that details the main limitations of your study.
***Reply:*** *Thank you for your suggestion. We have now modified the sentence. The text now states:”* *There is a low grade of evidence that cervical cerclage can prevent PTB in twin pregnancies. Emergency cerclage for cervical dilation or short cervical length <15 mm can be potentially associated with a reduction in PTB and improved perinatal outcomes. However, these findings are mainly based upon observational studies, thus limiting the robustness of the results, and require confirmation in large and adequately powered randomized controlled trials.”*

AUTHOR SUMMARY
At this stage, we ask that you include a short, non-technical Author Summary of your research to make findings accessible to a wide audience that includes both scientists and non-scientists. The Author Summary should immediately follow the Abstract in your revised manuscript. This text is subject to editorial change and should be distinct from the scientific abstract. Please see our author guidelines for more information: <https://journals.plos.org/plosmedicine/s/revising-your-manuscript#loc-author-summary>
***Reply:*** *Thank you for your suggestion. We have now provided the author summary. We hope it fits with the journal requests*

METHODS and RESULTS
Please update your search to the present time. We require that SRMAs are updated to within roughly 6 months of the expected publication date.

***Reply:*** *Thank you for your suggestion. We have now updated the search.*

Please provide the beginning and end dates of your search.
***Reply:*** *Thank you for your suggestion. This has been done now.*

Please also note the comments from the methodological reviewer (reviewer #1) regarding inclusion of non-English language sources of studies which we agree with. Please include non-English language sources of studies in your search.
***Reply:*** *Thank you for your comment. We have now updated the search. The initial search did not have any language restriction, but we decided to limit the inclusion of papers to those in English language. We have now re-run the search also considering studies not in English and we could not find any paper suitable for the inclusion .*

Thank you for reporting your SRMA according to PRISMA. Please remove the repeated statement at lines 132-133.
***Reply:*** *We apologize for the mistake. It has been corrected now.*

TABLES
To help facilitate transparent data reporting, PLOS medicine requests that where adjusted analyses are presented unadjusted analyses are also presented for comparison. We understand from your methods section that you extracted a combination of adjusted and unadjusted estimates from the studies included in your meta-analysis. We appreciate that this may not be feasible (or necessary) to present both in this case but it may be helpful if you could indicate which of the extracted data are adjusted and which are not and if the data are available might it also be helpful to include factors that were adjusted for? Perhaps in table 1?

***Reply:*** *Thank you for your comment. We have now added a column indicating which studies reported adjusted analyses and for which parameters the analyses were adjusted.*

When reporting p values please report as p<0.001 or where higher as p=0.002, for example. Please check and amend throughout including in the supporting files where relevant.
***Reply:*** *Thank you for your comment. We have now done that.*

SUPPORTING INFORMATION
Table S4 – is there a typo in column 3 (chorioamnionitis RCTs)?

***Reply:*** *Apologies for this mistake. We have now corrected all the tables.*

DISCUSSION
Please remove all sub-headings from the discussion such that it reads as a single piece of continuous prose starting with a short, clear summary of the articles findings; what the study adds to existing research and where and why the results may differ from previous research; strengths and limitations of the study; implications and next steps for research, clinical practice, and/or public policy; and ending in a one paragraph conclusion.
***Reply:*** *Thank you for your suggestion. All subheadings have been removed now.*

Comments from the reviewers:

Reviewer #1:

This paper describes a systematic review with meta–analysis of studies of cervical cerclage in twin pregnancies and its relationship with various adverse outcomes.

I was asked for a statistical report and I interpret that to include all aspects of the design and conduct of the study.

Points of detail

Page 2 Is it true that the pregnancies ‘were allocated to cerclage [. . . ]’? Few of the studies found here were trials where allocation is performed. The same wording is used elsewhere.
***Reply:*** *Thank you for your comment. You are right as only in a RCT the patients are allocated to given arm. We have now changed the sentence following your suggestion. The text now states:”* *Inclusion criteria were studies comparing the outcome of twin pregnancies at high risk of preterm birth, defined on the basis of short cervical length at ultrasound or cervical dilatation at digital examination, receiving compared to those not receiving cerclage”.*

Page 5 The section on language is not clear to me. Do the authors mean that they excluded all studies in a language other than English? The message I draw from Gr´egoire et al. (1995); Moher et al. (1996); Egger et al. (1997); J¨uni et al. (2002); Konno et al. (2020); Nussbaumer-Streit et al. (2020); Bachelet et al. (2021) is that bias is possible although it is hard to predict in advance the extent or direction of it. I would not necessarily commit the authors to translate everything but at least we should know what we are missing. (Jackson et al., 2019) has produced some evidence about the successful use of Google Translate in systematic reviews.

***Reply:*** *We entirely agree on the importance to include in the search also non-English written studies. Accordingly, please acknowledge that, also to comply with the Editor's request to update the literature search, we re-made it, this time with no language restriction. In the end, we came up with two new studies, both written in English, but no additional non-English study met the inclusion criteria.*

*Please also acknowledge that we clarified this issue in the methods section (page 5), where the sentence "The search and selection criteria were restricted to English language" has been cut, and the previous sentence "MEDLINE, Embase and CINAHL were searched electronically on the 6th July 2022 utilizing combinations of the relevant medical subject heading (MeSH) terms, keywords, and word variants for “twin pregnancies”, “multiple pregnancies”, “cerclage” and “preterm birth”" was rephrased as follows:*

*"MEDLINE, Embase and CINAHL were searched electronically on the 20th April 2023 utilizing combinations of the relevant medical subject heading (MeSH) terms, keywords, and word variants for “twin pregnancies”, “multiple pregnancies”, “cerclage” and “preterm birth”, with no language restrictions"*

Page 7 If authors were asked to supply extra data then I think that (a) we should be told which items we are not going to be able to ﬁnd in the original publications (b) the people who supplied it should be acknowledged.

*Reply: We agree on the need to clarify this issue, and we apologize for not having provided further details. Indeed, please acknowledge that authors were contacted with the aim of collecting extra-data on the different cut-offs of reduced cervical length (<15mm and 15-25mm), in case separate data were not extractable from the published studies, but, unfortunately, none of the contacted authors replied to our request. We clarified this issue on page 7, were the sentence "For those articles in which the relevant information was not reported but the methodology was such that the information might have been recorded initially, the authors were contacted, and the data requested" has been rephrased as follows: "For those articles in which data on reduced cervical length was not reported separately for subgroups of women (<15mm and 15-25mm), but the methodology was such that the information might have been recorded initially, the authors were contacted, and the data requested".*

Page 8 Conﬁdence intervals for I 2 would also be helpful to reveal our uncertainty about the heterogeneity. I know we did not use to use them but I am persuaded by Ioannidis et al. (2007) that we should.

***Reply:*** *We agree, and please acknowledge that all 95% CIs of the I2 have been computed using the heterogi command in Stata and added to the tables, where appropriate. Please also acknowledge that we mentioned this point in the data analysis section.*

Page 8 Since the various measures of risk are rather diﬀerent I would question the wisdom of comparing them. At least include a moderator for measure to account for diﬀerences.

***Reply:*** *We entirely agree and thank the Reviewer for the suggestion. Accordingly, please acknowledge that, also to comply with the Editor's request, the statistical approach has been substantially revised. First, all head-to-head meta-analyses now include only adjusted estimates (from non-randomized studies) and/or data extracted from randomized trials. Please also acknowledge that all analyses have been repeated (when possible) stratifying the data by study design (observational studies and RCTs). Additionally, please acknowledge that, given the presence of various measures of risk to be combined in most head-to-head meta-analyses (RRs in RCTs and adjusted ORs in observational studies), we converted all the adjusted ORs in RRs, adopting the approach described by Zhang et al (Journal of the American Medical Association, 280, 1690–91) and Hilbe (Logistic Regression Models, Chapman & Hall/CRC Press, 2008), and then re-computed all pooled estimates. Please also acknowledge that we mentioned this new approach in the data analysis section, were we added the following sentence "In case different measures of risk were to be included in the same pooled analysis (e.g. OR and RR), the OR was converted into RR adopting the approach described by Zhang et al (Journal of the American Medical Association, 280, 1690–91) and Hilbe (Logistic Regression Models, Chapman & Hall/CRC Press, 2008). Furthermore, we stratified all analyses according to the study design (randomized controlled trial or observational)".*

*Finally, please acknowledge that, to provide some estimates of the crude rates of each categorical outcome, we added meta-analyses of proportion, thus including in the analyses also the studies in which no adjusted estimates were available. The results of these new analyses have been reported in newly added, a dedicated table (Table 9), and they have been described in the data analysis section as follows: "Finally, in order to provide some estimates of the crude rates of each categorical outcome, we also performed meta-analysis of proportions, combining the data of women undergoing and not undergoing cerclage separately. To account for between-study heterogeneity, the analyses were performed using a random-effect model".*

Page 8 What funnel plots and regression tests hope to identify is small study eﬀects. Calling it publication bias implies you know the aetiology. Page 9 The authors have helpfully provided a copy of their data but it seems to only contain four randomised trials not ﬁve: Roman 2020, Berghellla 2004, Rust 2000, Althuisius 2001.

***Reply:*** *We entirely agree, and accordingly replaced the words "publication bias" with "small study effect" throughout the manuscript. We have also uploaded the data extraction files sued for the analyses.*

Page 10 I would suggest that the primary outcome should be reported ﬁrst not hidden in the middle of the secondaries. Page 11 onwards Some of these have lost their eﬀect size and its conﬁdence interval. Finding that the conﬁdence interval includes the null does not show no diﬀerence, it shows that the study was unable to show a diﬀerence.

***Reply:*** *We agree, and thank the Reviewer. Please acknowledge that the text was amended were appropriate. Please also acknowledge that the primary outcome was also reported on top of the others in the corresponding tables.*

Page 12 I am not sure what the relevance of comparing singleton pregnancies with twin pregnancies is. This needs clarifying.

***Reply:*** *Thank you for your comment. We have now deleted these analyses.*

Page 14 The authors state that ‘most of the included studies were not sufﬁciently powered’. Surely that is why we do meta–analysis, to draw conclusions from the totality of evidence?

***Reply:*** *We totally agree: accordingly, please acknowledge that the sentence "Furthermore, most of the included studies were not sufficiently powered to detect differences in the risk of the perinatal outcomes investigated" has been cut from the Discussion section.*

Figure 1 It would be better to clarify in this how many articles are relevant for the analysis of twin pregnancies and how many for the analysis of single versus twin.

***Reply:*** *Thank you for your comment. As reported above, we have now excluded these analyses.*

Points of more substance

Mixing trials and observational

I think most people would view trials as providing better information than observational studies not least in the avoidance of selection bias. The authors have not put forward a convincing justiﬁcation for presenting them mixed together. I took the data which the authors have kindly provided in machine–readable format and ran a meta–regression. I calculated the odds ratios from the ﬁgures for less than 34 weeks, the primary outcome. I included a moderator for study type and ﬁtted the model in R using the metafor package and estimating τ 2 with REML.. The relevant information was an eﬀect of 0.35 (95% ci 0.20 to 0.59) with a relative odds ratio for trials compared to observational of 5.50 (1.07 to 28.20). Since that does not include the null value (unity) I conclude that there is a real diﬀerence between the two study types with the observational studies being more optimistic. My conclusion would be that the observational studies are biased, probably because of selection bias perhaps caused by bias by indication. Obviously the authors will wish to conﬁrm my analysis with their own copy of the data and their software. Diﬀerent software often gives diﬀerent results.

***Reply:*** *We entirely agree, and thank the reviewer for his suggestions: accordingly, please find below the results of the meta-regression, performed through the REML approach, using the metareg command in Stata:*

```
------------------------------------------------------------------------------
       logrr |      Coef.   Std. Err.      t    P>|t|     [95% Conf. Interval]
-------------+----------------------------------------------------------------
      design |   .9296439   .8826002     1.05   0.323    -1.105636    2.964924
------------------------------------------------------------------------------
```

*In this case, the regression coefficient estimating the logRR of PTB<34th week in randomized versus observational trials does not show significant results, thus suggesting that there is not a real difference between the two study types. Please also acknowledge, however, that we entirely agree on potential problems arising from the combination of observational and randomized evidence. Accordingly, as described above in details, the analytical approach was substantially revised: (a) head-to-head meta-analyses included observational studies only when providing adjusted estimates; (b) RRs were re-computed from the adjusted ORs; finally (c) for all outcomes, stratified analyses were performed, separately evaluating randomized trials and observational studies, with most of the meta-analyses showing comparable pooled estimates.*

Small study eﬀects

The authors refer to the presence of small study eﬀects, for instance on page 13, although without presenting any evidence of them. Figure 1 shows the funnel plot from the meta–regression using study type as a moderator.

Note that with a model using a moderator the appropriate variable for the x–axis is the residuals from the model. In the plot I have marked the trials as triangles and the observational studies as circles. I do not see any noticeable evidence here of asymmetry bearing in mind the ﬁndings of Terrin et al. (2005) that researchers are poor at identifying asymmetry from funnel plots.

***Reply:*** *We entirely agree and thank the reviewer. Accordingly, please acknowledge that specific funnel plots (please see additional material for reviews) were added to the manuscript, with no noticeable evidence of asymmetry. We also entirely agree, however, on the potential limitation of a "visual" approach to detect asymmetry, and thus added, for each funnel plot, a quantitative evaluation performed through an Egger test: again, please acknowledge that no evidence of asymmetry emerged. Finally, given these results, please acknowledge that the previous mention to the potential "publication bias" on page 16 has been removed.*

Summary

As is clear I do not draw the same conclusions as the authors from their review. At the moment the trial evidence seems to be based on 78 pregnancies for the primary outcome. Given the conﬂict between those trials and the observational studies it would seem that what is needed is more trials. I have never been involved in an obstetric trial but I imagine the small number of participants reﬂects genuine diﬃculty in recruiting women into such trials but at the moment there must be genuine doubt about the advice they are to be oﬀered

***Reply:*** *Thank you for your comment. We entirely agree on the limited randomized evidence available (and on the scarce number of patients), which may be, inevitably, a potential limitation of the present meta-analysis. Please also acknowledge, however, that, following the Reviewer's comments (on which we totally agree), both the inclusion criteria and the methodological approach have been substantially revised, in order to be as conservative as possible, trying to decrease as much as possible the potential role of confounding factors in affecting the final estimates. We agree with you that the level of evidence is not enough to guide clinical practice. However, the purpose of a systematic review is not to provide and evidence but to elucidate whether there is evidence of a given topic. We think that the present systematic review, highlighting the major weaknesses and inconsistencies of previously published studies may serve as a basis to structure a RCT with more objective design and statistical power.*

References

V C Bachelet, M S Navarrete, C Barrera-Riquelme, V A Carrasco, M Dallaserra, R A D´ıaz, A A Ibarra, F J Lizana, N Mezza-Ducaud, M G Saavedra, C Tapia-Davegnoa, A F Vergara, and J Villanueva. A multiyear systematic survey of the quality of reporting for randomised trials in dentistry, neurology, and geriatrics published in journals of Spain and Latin America. BMC Medical Research Methodology, 21(153), 2021.

M Egger, T Zellweger-Z¨ahner, M Schneider, C Junker, C Lengeler, and G Antes. Language bias in randomised controlled trials published in English and German. Lancet, 350:326–329, 1997.

G Gr´egoire, F Derderian, and J Le Lorier. Selecting the language of the publications included in a meta-analysis: is there a tower of Babel bias? Journal of Clinical Epidemiology, 48(1):159–163, 1995.

J Ioannidis, N Patsopoulos, and E Evangelou. Uncertainty in heterogeneity estimates in meta–analyses. British Medical Journal, 335:914–916, 2007.

J L Jackson, A Kuriyama, A Anton, A Choi, J-P Fournier, A-K Geier, F Jacquerioz, D Kogan, and R Sun. The accuracy of Google Translate for abstracting data from non–English–language trials for systematic reviews. Annals of Internal Medicine, 171:677–679, 2019.

P J¨uni, F Holenstein, J Sterne, C Bartlett, and M Egger. Direction and impact of language bias in meta–analyses of controlled trials: empirical study. International Jounal of Epidemiology, 31:115–123, 2002.

K Konno, M Akasaka, C Koshida, N Katayama, N Osada, R Spake, and T Amano. Ignoring non–English–language studies may bias ecological meta–analyses. Ecology and Evolution, 10:6373–6384, 2020.

D Moher, P Fortin, A R Jadad, P J¨uni, T Klassen, J Le Lorier, A Liberati, K Linde, and A Penna. Completeness of reporting of trials published in languages other than English: implications for conduct and reporting of systematic reviews. Lancet, 347:363–366, 1996.

B Nussbaumer-Streit, I Klerings, A I Dobrescu, E Persad, A Stevens, C Garrity, C Kamel, L Aﬀengruber, V J King, and G Gartlehner. Excluding non–english publications from evidence–syntheses did not change conclusions: a meta–epidemiological study. Journal of Clinical Epidemiology, 118:42–54, 2020.

N Terrin, C H Schmid, and J Lau. In an empirical evaluation of the funnel plot, researchers could not visually identify publication bias. Journal of Clinical Epidemiology, 58:894–901, 2005.

***Reviewer #2***:

The paper presents a systemic review and meta-analysis of cervical cerclage for prevention of preterm birth and adverse perinatal outcome in twin pregnancies. The description of the study rationale, the interpretation of the results and the discussion are balanced.

***Reply:*** *Thank you for these positive comments on the study.*

However, similar articles have been published in the peer-reviewed scientific literatures such as,

Su J, Li D, Yang Y, Cao Y, Yin Z. Cerclage placement in twin pregnancies with cervical dilation: a systematic review and meta-analysis. J Matern Fetal Neonatal Med. 2022 Dec;35(25):9112-9118. doi: 10.1080/14767058.2021.2015577. Epub 2021 Dec 14. PMID: 34906023.

Li C, Shen J, Hua K. Cerclage for women with twin pregnancies: a systematic review and metaanalysis. Am J Obstet Gynecol. 2019 Jun;220(6):543-557.e1. doi: 10.1016/j.ajog.2018.11.1105. Epub 2018 Dec 7. PMID: 30527942.

Li C, Hua K. Efficacy of physical examination-indicated cerclage in twin pregnancies compared with singleton pregnancies: a systematic review and meta-analysis. Minerva Obstet Gynecol. 2021 Feb;73(1):111-120. doi: 10.23736/S2724-606X.20.04518-9. Epub 2020 Apr 21. PMID: 32315128.

Saccone G, Rust O, Althuisius S, Roman A, Berghella V. Cerclage for short cervix in twin pregnancies: systematic review and meta-analysis of randomized trials using individual patient-level data. Acta Obstet Gynecol Scand. 2015 Apr;94(4):352-8. doi: 10.1111/aogs.12600. Epub 2015 Mar 1. PMID: 25644964.

The authors should mention the previous studies and discuss the additional information of this manuscript compared with previous studies.

***Reply:*** *Thank you for your comment. We have now provided a detailed explanation of the strengths of the present systematic review compared to what has been previously published. Essentially, we included a higher number of studies (most of the studies on cerclage in twins has been published in the last two years, after these reviews were published), the multitude of outcomes explored, the stratification of the analysis according to different cut-offs of cervical length and the assessment of the explored outcomes according to the study deign. The text now states:”* *This is, to the best of our knowledge, the largest and most up to date systematic review and meta-analysis on the role of cervical cerclage in affecting PTB in twins. Previous systematic reviews have addressed the association between cerclage and perinatal outcome in twins [12, 48-50]. Compared to this review, the present study, includes a well specific population of twin pregnancies at high-risk for PTB, defined on the presence of a short cervical length at ultrasound or cervical dilatation at physical examination, a large number of outcomes explored, stratification of the analyses according to cervical length at ultrasound or cervical dilatation and computation of the observed outcome according to the study design (observational vs RCT)”.*

Besides, PPROM should be preterm prelabor rupture of membranes (ACOG Practice Bulletin No. 217: Prelabor Rupture of Membranes. Siegler Y, et al. Obstet Gynecol. 2020. PMID: 33093409).
***Reply:*** *We apologize for this mistake. We have now corrected it.*

Reviewer #3: This systematic review addresses the important question of cerclage in twins. Historically cerclage has been said to be contraindicated, and this was based on a small meta analysis by Berghella that was under powered to inform on the rarer women with a very short cervical length. Indeed, in the UK, NICE, as an arbitor of clinical practice, until recently specifically advised against cervical scanning in women with twins. More recently a number of small series or trials in various heterogeneous groups have presented data that challenges this.

A synthesis of the results looks at
1. Overall, &apos;in pregnancy&apos; cerclage (<25mm or physical exam), or whatever the authors wish to call it, but not emergency cerclage.
Subgroups of indication for &apos;in pregnancy&apos; cerclage:
2. Cervical length <15 mm
3. Physical examination
Then:
4. Pessary (without conclusions)
5. Twins versus singletons
This is a highly appropriate paper and should be published.
***Reply:*** *Thank you for the positive comments on the study.*

Points.
1. I am not an expert in the methodology and so am not qualified to comment on this.
2. The authors have confused me on definitions. They define emergency cerclage as that where the cervix is <25mm or physical examination. This is not correct by conventional terminology. It is not unreasonable to group these together but this perhaps should be defined as non-elective or &apos;short cervix/ physical examination&apos;.

***Reply:*** *Thank you for this comment. We have now included in the present systematic review and meta-analysis, a well specific population of twins, defined as those with short cervical length at ultrasound and those with cervical dilatation. We have also provided different analyses according to the ultrasound cut-off of cervical length and according to the study design, as requested by the editor.*

3. Even though putting cervical cerclage at 0mm cervical length, short cervical length +/- with bulging membranes together with &apos;physical examination indicated&apos; is pragmatic, it is still a bit problematic because of the heterogeneity: the US RCT of physical examination indicated cerclage (Roman et al) did not specify cervical length in all women and some were clearly not that short. The heterogeneity of this group should be emphasised, and perhaps a later comment about the different practice in Europe (cervical length) and the US (physical examination)

***Reply:*** *Thank you for this comment. We have now stressed the limitations of the included populations and also highlight the need for RCT with well specific indications for cerclage. The text now states: “Although the findings from this meta-analysis suggest a potential beneficial role of cervical cerclage in reducing the risk of PTB and improving neonatal outcome in women at risk, the inclusion of mainly observational studies significantly affect the robustness of the results and should be confirmed in adequately powered RCTs. Only three RCTs were included, with a very small number of women allocated to cerclage or standard care. Ideally, a RCT on the role of cerclage in twin pregnancies should include women with short cervix on ultrasound or cervical dilatation separately and be powered to maternal and neonatal outcomes. Furthermore, this trial should share an objective protocol for prenatal assessment of women at risk and management of women before and after cerclage placement, including timing of ultrasound assessment of cervical length by ultrasound and preventive strategies for PTB, including progesterone and tocolysis.”*

4. Line 302:;Conversely, cerclage in women with a cervical length between 5 and 25 mm was not.. &apos; This is a typo? Should 5 not be 15?

***Reply:*** *We apologize for this mistake. It has been corrected now.*

5. I would like to see data in the text as to effect sizes particularly whether any outcomes were increased (as opposed to not reduced) in the 15-25mm cervical length group.

***Reply:*** *Thank you for this comment. We have now reported the analyses for different cut-offs of cervical dilatation. Although the numbers are small, the data show a beneficial effect in women with CL <15 mm but not between 15 and 25 mm*

6. Could the authors differentiate between monochorionic and dichorionic twins?

***Reply:*** *Thank you for this comment. Unfortunately, a pooled data analysis on monochorionic and dichorionic twins was not feasible.*

7. Given the importance of the findings and the need for evidence-based practice I would like to see, in the conclusions, a recommendation regarding best practice, including recommending against cervical cerclage where the cervix is >25mm.
***Reply:*** *Thank you for this suggestion. We have now stressed this recommendation. The text now states:”* *On this basis, placement of cervical cerclage in women with cervical length on ultarsound >15 mm, should not be considered, although these findings are based upon a non-randomised evidence”.*

Reviewer #4: The authors present a meta-analysis of both observational and randomised control trials of cerclage in twins. This is in a currently challenging area with considerable uncertainty and variation in clinical practice. The topic is important and worthy of publication. However we believe that more data could be derived from their analysis that would have important clinical implications.
***Reply:*** *Thank you for this comment. We have now re-analyses all outcomes and stratified the analysis according to the type of study. We hope that you will find the paper improved.*

The authors have subdivided their groups for analysis into two main categories:
Those with dilated cervix and/or those with a shortened cervix <25mm
Elective cerclage (who have presumably not got a short cervix, but this is not clear)

Those in the first group may be substantially different, depending on whether the cervix id dilated or not, and cerclage could have varying treatment affects in these groups. A dilated cervix with exposed membranes will allow immediate ascending infection and a cerclage in effect is trying to reverse a high risk situation. However the group with a cervix <25mm may be upstream in the pathophysiological process and a cerclage is more preventative. It is unclear why they have combined these groups. Indeed their sub-analysis demonstrates a difference in those with cervix >15mm and <15mm, suggesting the concerns above are valid. We are a little concerned that this combination may have been performed to avoid a type II error in one of these sub-groups.
***Reply:*** *Thank you for this comment. We have combined studies according to the primary indications for cerclage, including those with short cervix at ultrasound and those with cervical dilatation. We agree with you that women with cervical dilatation may be at higher risk of cerclage failure and adverse maternal and perinatal outcomes. However, a comparison between cerclage for short cervical length at ultrasound vs cervical dilatation could not be performed as there was no study comparing the tow indications for cerclage, making the computation of pooled risk ratio not possible.*

We strongly recommend primary analysis is performed separately for women with dilated cervix and in those with a short cervix, as well as those with an elective procedure (i.e. three groups in total). Or alternatively reasons for not doing this described.
***Reply:*** *Thank you for this comment. We have now provided separate analyses on all the explored outcomes in women with short cervix (also stratified by the degree of cervical length) and cervical dilatation.*

An additional analysis which would be clinically very relevant would be those with symptomatic and asymptomatic presentations. Outcomes following emergency cerclage are very different if women present with discharge and pain at presentation and subsequently had a cerclage versus those who were incidentally found to have a short cervix. We don&apos;t feel as strongly about this point but some exploratory analysis whether this clinical factors are important, should they be available in their data, would be worthwhile.
***Reply:*** *Thank you for this comment. Unfortunately, this analysis could not be performed. All studies included asymptomatic women. Placement of cerclage in women with symptoms (i.e. uterine contractions) is not recommended. Likewise, there was no data on symptoms like discharge.*

The terminology used for elective cerclage is unselected populationions;. This is not a commonly used terminology and is ambiguous as it implied all comers including those with a short cervix, etc. Again, this should be clarified and we believe the term is elective cerclage is more ubiquitous.
***Reply:*** *Thank you for this comment. As also suggested by the Editor, we have now deleted the analyses on unselected twins.*

In the group with elective cerclage, a problem with all research in twin pregnancies is that low risk women are often recruited, as they are easy to obtain for clinical trials. However, are there are sound pathophysiological reasons why women with risk factors may behave differently. At very least they should discuss if studies evaluated these sub-groups and ideally perform a separate analysis if the data if the data is available.
***Reply:*** *Thank you for this comment. As also suggested by the Editor, we have now deleted the analyses on unselected twins.*

A general comment is that it may be wise to improve the readability of the article, as we must confess to having to read several paragraphs multiple times. We appreciate the challenge of writing in a language that is not one&apos;s native language, however the team will likely have the expertise to improve this.
***Reply:*** *Thank you for this comment and we apologize for not being clear and concise. We have now extensively revised the English.*

This is an important piece of work and the team have done well with good analysis and correct endpoints. We believe with extra analysis, significantly more clinically relevant findings could be obtained (if the data is available). We strongly recommend this with a view to publication.

Reviewer #5: nicely written concise systematic review with sound methodology and appropriately utilised tools for the meta-analysis.

***Reply:*** *Thank you for these positive comments on the study.*

Reviewer #6: This paper is confusing.
My main concern is that the authors pool everything together and this does not facilitate using this paper in clinical practice (see also comments below).

***Reply:*** *Thank you for your comment. We have now extensively revised the study. Inclusion criteria are twin at risk of PTB defined upon the presence of cervical dilatation or short cervix on ultrasound. We have therefore deleted the analysis on unselected twins and the comparison of cerclage in twins vs singleton. We have carry all the analyses again separating studies reporting comparing to those not reporting adjusted estimated bases upon prior PTB, parity and other risk factors for PTB. We have also provided new analyses reporting both the primary and secondary outcomes in RCT and observational studies separately. We think that the paper is improved now. We would also like to stress the fact that the aim of a systematic review is not to provide an evidence but show whether evidence is present on a given topic in order to design better RCTs.*

They pool cases with an ultrasound indicated cerclage with those of physical examination indicated cerclage in patients with cervical dilatation, generally referred to as &apos;emergency cerclage;. I advise you to differentiate between those 2 groups.

***Reply:*** *thank you for your comment. We have provided separate analyses for women with twin pregnancies undergoing cerclage based upon a short cervix or cervical dilatation separately.*

Moreover, as stated in sentence 136 and 137: inclusion criteria were studies in which twin pregnancies were allocated to cerclage for the prevention of PTB or to a control group (e.g. placebo or treatment as usual). Thus the studies comparing with pessary should not be included, this is confusing.

***Reply:*** *thank you for your comment. We have now excluded studies with pessary.*
