# Supplementary material for: Cervical cerclage for prevention of preterm birth and adverse perinatal outcome in twin pregnancies with short cervical length or cervical dilatation: A systematic review and meta-analysis
Source: PLoS Med. 2023 Aug 3;20(8):e1004266. doi: 10.1371/journal.pmed.1004266 (PMC10456178; doi:10.1371/journal.pmed.1004266)
Supplement: Attachment — Submitted filename: Reply_to_reviewers.docx [file pmed.1004266.s015.docx]

Requests from Editors:

GENERAL
Thank you for your detailed and considered responses to previous editor and reviewer comments.
Please see below for further comments which we require you address in full, prior to publication.
Throughout, please replace the term ‘retrospective’ with ‘observational’ when describing your study.
***Reply:*** *Thank you for your positive comments. We have now modified the manuscript following your suggestions.*

STATISTICAL REPORTING
Suggest separating upper and lower bounds of CIs with commas as opposed to hyphens which can be confused with reporting of negative values.
PLOS requests that where 95% CIs are reported p values are also reported, please include. Please report p values as <0.001 and where higher as p=0.002, for example.
Suggest reporting statistical information as follows, ‘(RR: 0.73, 95% CI [0.59,0.91], p=/<)’ for improved reader accessibility. Please check and amend throughout the main manuscript, tables and figures where relevant, including in the supporting information.

***Reply:*** *Thank you for your comment. This has been done now.*

ABSTRACT
Line 47 – please expand the background to (briefly) provide wider context of why the study question is important. The statement currently written at line 47 should constitute the last sentence of the Abstract

***Reply:*** *Thank you for your comment. This has been done now.*

Background.
Line 51 – thank you for updating your search. Please include the search start date – e.g. inception or a specific date and in the event that you report a specific date please briefly justify the reasons for its choice.

***Reply:*** *Thank you for your comment. We have now added this information to the search.*

Line 65 onward - please include the actual amounts and/or absolute risk(s) of relevant outcomes, not just relative risks or correlation coefficients (example for absolute risks: PMID: 28399126).
***Reply:*** *Thank you for your comment. We have now added this information to the search.*

Line 72 onward – as above, suggest separating upper and lower bounds of CIs with commas as opposed to hyphens which can be confused with reporting of negative values. PLOS requests that where 95% CIs are reported p values are also reported, please include. Please report p values as <0.001 and where higher as p=0.002, for example.

***Reply:*** *Thank you for your comment. It has been done now.*

Line 74 – please define MD (? Mean difference) prior to first use here – perhaps at line 66?
***Reply:*** *Thank you for your comment. We have now defined MD.*

AUTHOR SUMMARY
Thank you for including an author summary. Please see below for suggested revisions to formatting and minor changes to the content:

Why Was This Study Done?
* Twin pregnancies are at higher risk of preterm birth (PTB).
* Recent evidence suggests that placement of cervical cerclage in twin pregnancies with short cervical length at ultrasound or cervical dilatation at physical examination might be associated with a reduced risk of PTB.
* However, such evidence is based mainly on small studies thus questioning the robustness of these findings.

What Did the Researchers Do and Find?
* We performed a systematic review and meta-analysis to elucidate whether cervical cerclage in women with twin pregnancy with short cervical length or cervical dilatation may prevent PTB. We included eighteen studies. The primary outcome was PTB <34 weeks of gestation.
* We found that cervical cerclage in women with short cervical length or cervical dilatation was associated with a reduced risk of PTB <34 weeks, gestational age at birth and adverse neonatal outcome.
* The strength of association between cerclage and reduced risk of PTB was maintained when considering women with short cervix on ultrasound and those with cervical dilatation at physical examination separately.

What Do These Findings Mean?
* Cervical cerclage in twin pregnancies with short cervical length or cervical dilatation may be potentially associated with a reduced risk of PTB and improved neonatal outcomes.
* However, these findings are mainly based on observational studies and, to improve robustness of evidence, confirmation of these outcomes in large and appropriately designed RCTs is required.
***Reply:*** *Thank you for your suggestion. We have now modified this part of the manuscript following your suggestions.*

METHODS and RESULTS
As for the abstract please include details of the beginning dates of your search.
Line 192 – ‘(McDonald vs Shirodkar)’ please indicate for the reader that these are different techniques and the most utilised approaches for cerclage.

***Reply:*** *Thank you for your suggestion. This information has been added to the methods now.*

Line 252-3 – please apply PLOS Medicine’s required referencing format here.

***Reply:*** *Apologies for this mistake. It has been corrected now.*

Line 267 onward – please use either numbers or words to depict numerical values as opposed to a combination of both.

***Reply:*** *Apologies for this mistake. It has been corrected now.*

Lines 288-290 – see above under statistical reporting. Here you use the word ‘to’ to separate upper and lower bounds in view of negative values, suggest instead using commas throughout.

***Reply:*** *Thank you for your suggestion. We have now modified the manuscript following your suggestions.*

Line 293 onward – please amend statistical reporting as detailed above. Please check and amend throughout.

***Reply:*** *Thank you for your suggestion. We have now modified the manuscript following your suggestions.*

Line 308 – ‘pPROM’ please ensure this has been defined at first use for the reader – apologies if I have missed it.
***Reply:*** *Thank you for your comment. pPROM has been defined now.*

TABLES
Table 1 – column ‘Gestational age at cerclage placement’ please define the age measurements (weeks/days). Please define the numerical values contained within parentheses. Does the +/- refer to days or weeks or both? Throughout, please define ‘NR’ for the reader. Please replace ‘retrosp.’ with ‘OBS’ or similar to describe the studies as observational and please define the abbreviation in the caption for the reader.

***Reply:*** *Thank you for your suggestion. We have now add “weeks” to gestational age, replaced retrosp with observational and report and explanation for NR (not reported)*

Table 3 – please indicate if more (or less) stars equate to higher (or lower) quality.

***Reply:*** *Thank you for comment. It has been done now.*

Table 4 – throughout where reporting 95% CIs please separate upper and lower bounds with commas (not semi-colons or hyphens). Is there room to write ‘Number of studies’ in the column 2 header?

***Reply:*** *Thank you for comment. It has been done now.*

Please change the penultimate column header to ‘p value’.
***Reply:*** *Thank you for comment. It has been done now.*

Table 5, 6, 7 & 8 – as above

***Reply:*** *Thank you for comment. It has been done now.*

DISCUSSION
Line 466 – please remove the sub-heading ‘Conclusions’

***Reply:*** *Thank you for comment. It has been done now.*

Line 474 – please remove the funding statement and include only in the manuscript submission form when you resubmit the manuscript, it will be compiled as metadata at the time of publication.
***Reply:*** *Thank you for comment. It has been done now.*

REFERENCES
Ref 28 – appears incomplete
***Reply:*** *Apologies for the mistake. It has been corrected now.*

SUPPORTING INFORMATION
Thank you for including the PRISMA checklist. Please revise to refer to section and paragraph numbers as opposed to page (and/or line) numbers as these often change at publication.

SOCIAL MEDIA
If not already done so, to help us extend the reach of your research, please detail any Twitter handles you wish to be included when we tweet this paper (including your own, your coauthors’, your institution, funder, or lab) in the manuscript submission form when you re-submit the manuscript.
***Reply:*** *Thank you for your request. Here my university (university of Chieti) twitter account:* [*https://twitter.com/univuda?lang=en*](https://twitter.com/univuda?lang=en)*; mine:*

Comments from Reviewers:

Reviewer #1: The authors have addressed all my points.

***Reply:*** *Thank you.*

Michael Dewey

Reviewer #3: The heterogeneity of studies included (observational and randomised; physical exam indicated, emergency and cervical length/ different cervical lengths) were always going to make this analysis difficult and limit its conclusions.
The authors have performed considerable work to address the issues raised by the multiple reviewers and I believe have done as good a job as is possible. Although I remain slightly concerned about the different conclusions regarding observation and randomised studies with the statistical reviewer 1, I am not convinced this matters too much as the conclusions are clear that the positive findings apply to observational rather than randomised studies. This limits their robustness but again the authors are clear about this.
***Reply:*** *Thank you for your positive comments. We think we have now stressed the limitations of the results better than the first version.*

Reviewer #6: Nicely performed systematic review. I am satified with the modifications made.
***Reply:*** *Thank you for your positive comments.*
